# USP18 enhances dengue virus replication by regulating mitochondrial DNA release

**DOI:** 10.1038/s41598-023-47584-w

**Published:** 2023-11-17

**Authors:** Jenn-Haung Lai, De-Wei Wu, Chien-Hsiang Wu, Li-Feng Hung, Chuan-Yueh Huang, Shuk-Man Ka, Ann Chen, Ling-Jun Ho

**Affiliations:** 1 Department of Rheumatology, Allergy and Immunology, Department of Internal Medicine, Chang Gung Memorial Hospital, Lin-Kou, Tao-Yuan, Taiwan, ROC; 2 Institute of Cellular and System Medicine, National Health Research Institute, Zhunan, Taiwan, ROC; 3 Graduate Institute of Aerospace and Undersea Medicine, Department of Medicine, National Defense Medical Center, Taipei, Taiwan, ROC; 4 Department of Pathology, Tri-Service General Hospital, National Defense Medical Center, Taipei, Taiwan, ROC

**Keywords:** Immunology, Diseases

## Abstract

Dengue virus (DENV) infection remains a challenging health threat worldwide. Ubiquitin-specific protease 18 (USP18), which preserves the anti-interferon (IFN) effect, is an ideal target through which DENV mediates its own immune evasion. However, much of the function and mechanism of USP18 in regulating DENV replication remains incompletely understood. In addition, whether USP18 regulates DENV replication merely by causing IFN hyporesponsiveness is not clear. In the present study, by using several different approaches to block IFN signaling, including IFN neutralizing antibodies (Abs), anti-IFN receptor Abs, Janus kinase inhibitors and IFN alpha and beta receptor subunit 1 (IFNAR1)knockout cells, we showed that USP18 may regulate DENV replication in IFN-associated and IFN-unassociated manners. Localized in mitochondria, USP18 regulated the release of mitochondrial DNA (mtDNA) to the cytosol to affect viral replication, and mechanisms such as mitochondrial reactive oxygen species (mtROS) production, changes in mitochondrial membrane potential, mobilization of calcium into mitochondria, 8-oxoguanine DNA glycosylase 1 (OGG1) expression, oxidation and fragmentation of mtDNA, and opening of the mitochondrial permeability transition pore (mPTP) were involved in USP18-regulated mtDNA release to the cytosol. We therefore identify mitochondrial machineries that are regulated by USP18 to affect DENV replication and its association with IFN effects.

## Introduction

Dengue virus (DENV) infection is a critical public health concern worldwide^1^. The incidence of potentially fatal consequences of DENV infection, such as dengue hemorrhagic fever (DHF) and dengue shock syndrome (DSS), has not notably changed in recent decades^2, 3^. It is estimated that 1 in 20 DENV-infected patients will suffer severe consequences, such as shock, internal bleeding and death^4^. The aggressive development of anti-dengue vaccines is currently underway, but these efforts still face great challenges^4, 5^.

Ubiquitin-specific protease 18 (USP18), also known as UBP43, is an IFN-inducible molecule preserving isopeptidase activity that deconjugates proteins by targeting interferon-stimulated gene 15 (ISG15); this process is called deISGylation^6^. The deISGylation process helps to protect ISG15-ISGylated target proteins from degradation via an autophagy-dependent pathway^7^. After recruitment by signal transducer and activator of transcription (STAT) 2, USP18 interacts with interferon alpha and beta receptor subunit 2 (IFNAR2) through its C-terminal domain and competes with Janus kinase (JAK)1 for binding and transducing signals to inhibit type I IFN (IFN-I)-mediated effects^8, 9^. Thus, USP18 serves as the potential target that is used by DENV to antagonize the antiviral effects of IFNs and to achieve immune evasion^10^. Studies have shown that the negative effects of USP18 on IFN-I may be beneficial for preventing the overactivation of immune responses in autoimmune diseases^11^. In a model of vesicular stomatitis virus (VSV) infection, Honke et al.^8^ demonstrated that by lowering IFN-I responsiveness, USP18 sustained viral replication in the local milieu to ensure the production of sufficient viral antigen for the effective induction of adaptive immunity. However, whether the reported effects of USP18 occur simply through anti-IFN signaling remains incompletely understood.

Mitochondria function as pivotal organelles for triggering antiviral immunity and, at times, may be targeted by viruses to evade immune surveillance^12^. As a molecule that is localized to mitochondria, how USP18 may regulate mitochondrial machinery to affect DENV replication has not been investigated. In here, we addressed (1) whether USP18 regulates DENV replication in a manner that is completely dependent on IFN signaling inhibition and (2) how USP18 may regulate DENV replication by affecting the release of mtDNA, a mechanism driving toll-like receptor 9 (TLR9) activation as reported by our group^13^. The results suggest that USP18 regulates DENV replication through both IFN-associated and IFN-unassociated mechanisms. By modulating mitochondrial machineries, USP18 regulates mtDNA release to the cytosol to affect DENV replication.

## Results

### DENV infection induced USP18 which regulated DENV replication through in part IFN-independent manner

**Figure 1:**
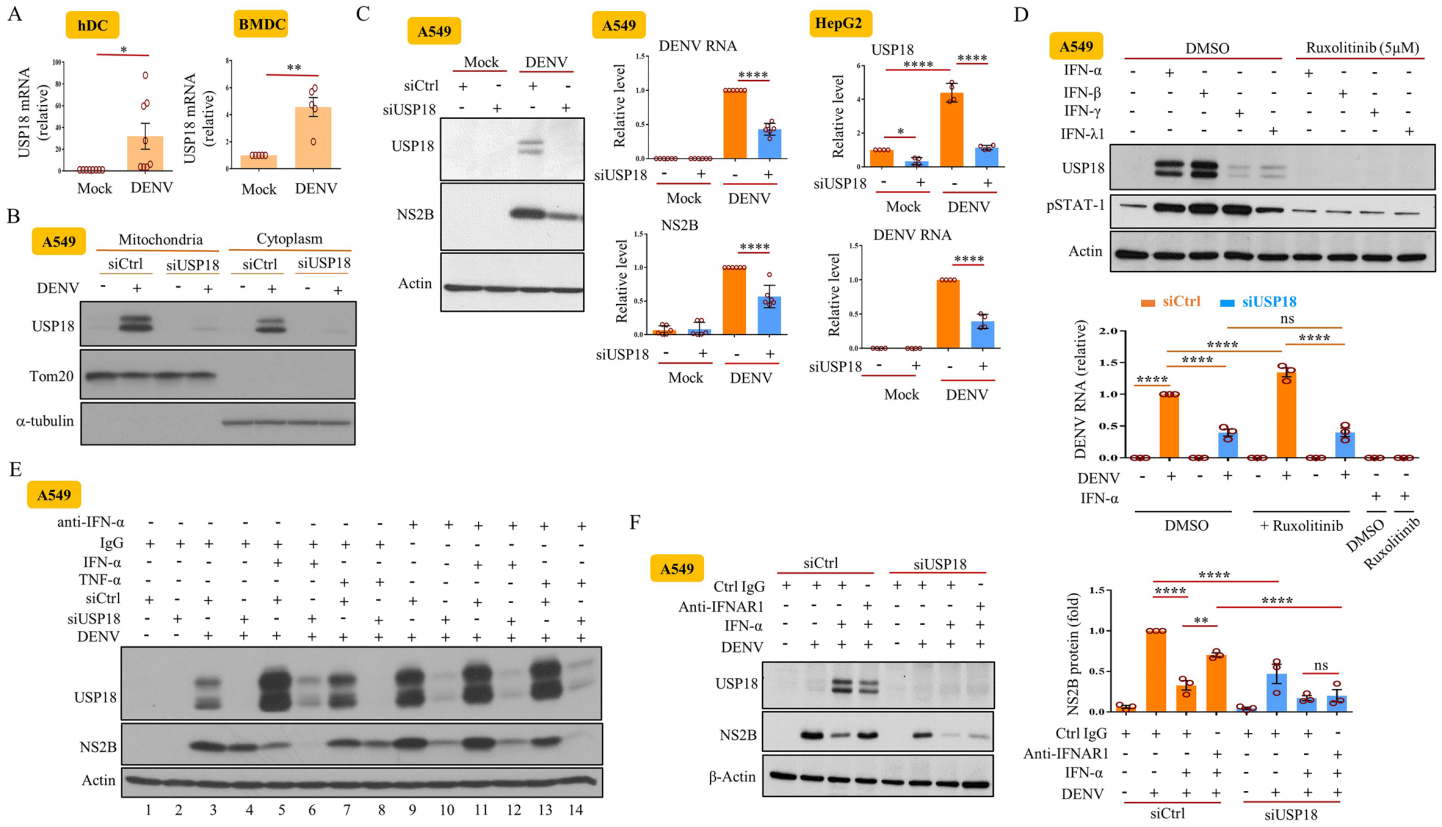
DENV infection induced USP18 that regulated DENV replication through in part IFN-independent manner. Human dendritic cells (hDCs) and mouse bone marrow-derived DCs (BMDCs) (5 × 10^6^/ml) were infected with DENV (MOI = 5 for hDCs and MOI = 0.5 for BMDCs) or mock for 24 h, and the expression of USP18 mRNA was determined by qPCR (**A**). A549 cells (2 × 10^5^/ml) were introduced with siUSP18 or control siRNA (siCtrl) via lipofectamine 3000 (3 μl/ml) according to the description by the manufacturer. After this, cells were infected with DENV (MOI = 0.5) or mock for 24 h, and the expression of USP18, Tom20 and α-tubulin in cytosolic and mitochondrial fractions was determined by Western blotting (**B**). Similar to (**B**), the effects of USP18 deficiency (siUSP18 treatment) on DENV NS2B and DENV RNA levels measured by Western blots and qPCR in A549 cells and HepG2 cells were determined (**C**). A549 cells (2 × 10^5^/ml) were treated with 5 μM ruxolitinib for 2 h and then stimulated with various IFNs (IFN-α (100 U/ml), IFN-β (50 U/ml), IFN-γ (10 ng/ml), and IFN-λ1 (40 ng/ml)), and the expression of USP18 and phosphorylated STAT1 was determined by Western blotting (**D**, upper). Similarly, effects of ruxolitinib on USP18 deficiency-regulated DENV RNA levels measured by qPCR were evaluated (**D**, lower). A549 cells (2 × 10^5^/ml) treated with siCtrl or siUSP18 were pretreated with anti-IFN-α neutralizing Abs (5 μg/ml) or control Abs for 2 h and then stimulated with IFN-α (100 U/ml) or TNF-α (40 ng.ml), followed by infection with mock or DENV (MOI = 0.5) for 24 h, and the expression of mRNA of USP18 and DENV NS2B was measured (**E**). The effects of anti-IFNAR1 neuralizing Abs (5 μg/ml) on DENV-induced USP18 and NS2B expression with or without USP18 deficiency were measured (**F**). More than 3 independent experiments were analyzed for each condition. Statistical analysis was done using Student’s t test (**A**, **C**) and two-way ANOVA with Holm-Sidak (**D**, **F**) to compare differences among different treatments. *P < 0.05; **P < 0.01; ***P < 0.001 and ****P < 0.0001. Original gels are presented in Supplementary Fig. 9.

**Figure 2:**
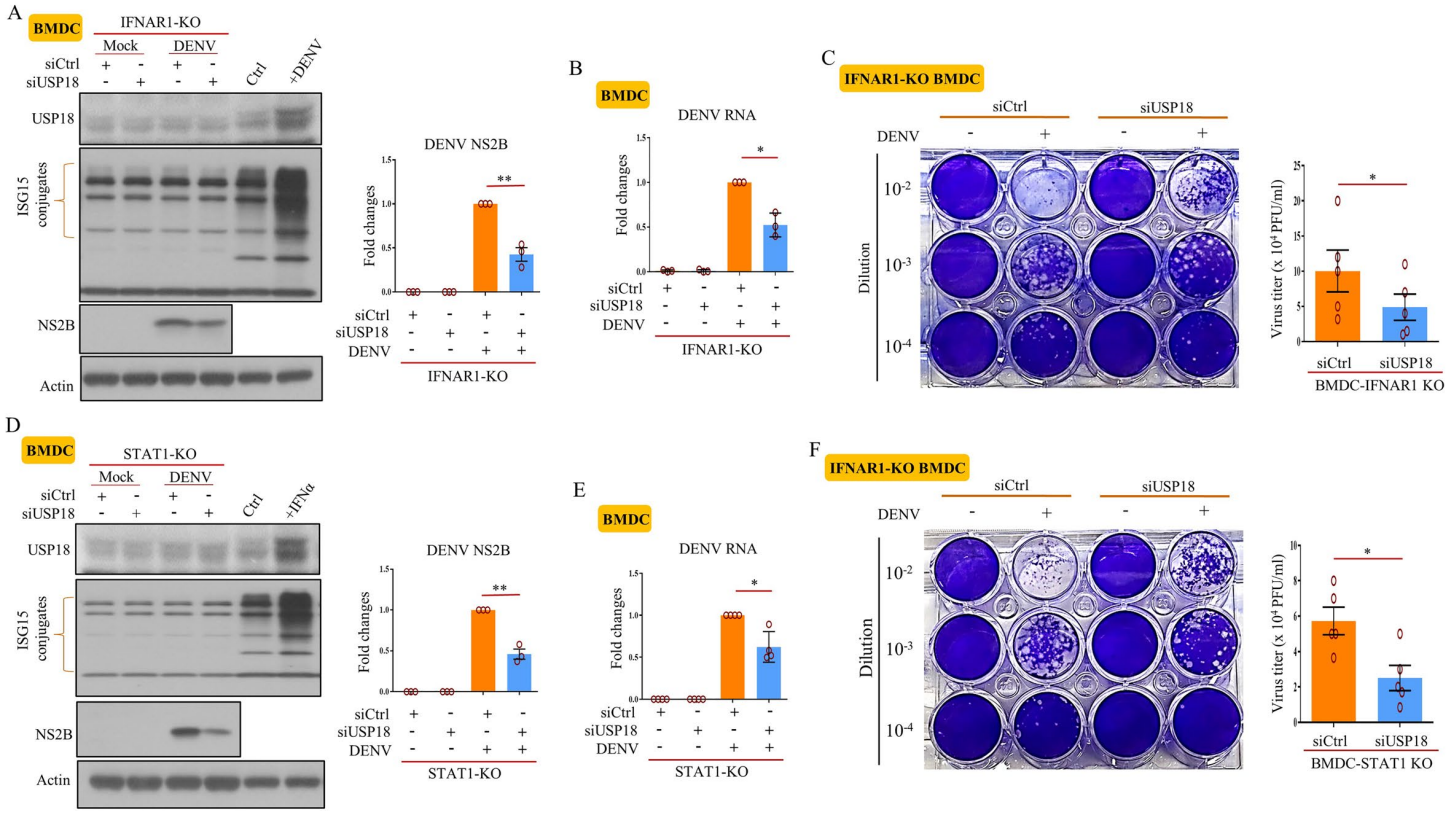
USP18 deficiency inhibited DENV replication in BMDCs from *ifnar1*-KO and *stat1*-KO mice. BMDCs were prepared from *ifnar1*-KO mice as described in the Materials and Methods. After introduction of siCtrl or siUSP18 via electroporation as described in the Materials and Methods, the cells (1 × 10^6^/ml) were infected with mock or DENV (MOI = 1) for 24 h and then collected for further analysis. The expression of USP18 and NS2B, and protein ISGylation in total cell lysates was determined by Western blotting (**A**). The levels of DENV RNA were measured by qPCR (**B**). In addition, the DENV titers were evaluated by plaque assays as described in the Materials and Methods (**C**). Similar experiments were carried out in BMDCs from *stat1*-KO mice and the results were presented in (**D**, **E** and **F**), respectively. Values represent the mean of the individual measurements in each sample ± SEM. Statistical analysis was done using Student’s t test (**A**–**F**) to compare differences among different treatments. *P < 0.05, **P < 0.01. Original gels are presented in Supplementary Fig. 10.

Microarray analysis revealed that USP18 was one of the genes that were upregulated in hDCs infected with DENV (Supplementary Fig. 1A). All required messages about the microarray data were included in our recently published report^14^. USP18 induction also occurred in primary BMDCs infected by DENV (Fig. 1A). DENV infection-induced USP18 expression and protein ISGylation depended on IFNAR and STAT1 (Supplementary Fig. 1B,C). To investigate the mechanism of USP18-regulated DENV replication and its association with IFN, USP18 expression was attenuated by introducing USP18 small interference RNA (siUSP18) into cells. The Fig. 1B showed that DENV infection induced the accumulation of USP18 in mitochondria that was dramatically reduced by siUSP18 treatment. USP18 deficiency inhibited DENV replication in different tissue cells (Fig. 1C). We used several approaches to determine whether USP18 could regulate DENV replication independently of its anti-IFN effects. The JAK1/2 inhibitor ruxolitinib, which completely blocked IFN-α-, IFN-β-, IFN-λ1- and IFN-γ-induced STAT1 phosphorylation, enhanced DENV replication, but had no effects on USP18 deficiency-mediated reduction of DENV replication (Fig. 1D). To more specifically target IFN, the effects of both anti-IFN-α neutralizing Abs and anti-IFNAR1 Abs were examined. While anti-IFN-α Abs completely blocked the IFN-α-mediated inhibition of viral production, it had limited effects on the USP18 deficiency-mediated inhibition of viral production (Fig. 1E, compare lane 11 with 5 and lane 12 with 6); TNF-α treatment served as a control in this experiment. To show the adequacy of the anti-IFN effects by different treatments, we added IFN-α into cell cultures in all experiments that were conducted to study the effects of USP18 deficiency. We also observed that anti-IFNAR1 Ab treatment could not reverse the USP18 deficiency-mediated suppression of DENV replication (Fig. 1F). Furthermore, USP18 deficiency significantly attenuated the expression of DENV NS2B protein, DENV RNA and DENV viral particles in BMDCs derived from mice lacking the *ifnar1* (Fig. 2A–C) and *stat1* genes (Fig. 2D–F).

### USP18 regulated mtDNA release to the cytosol

**Figure 3:**
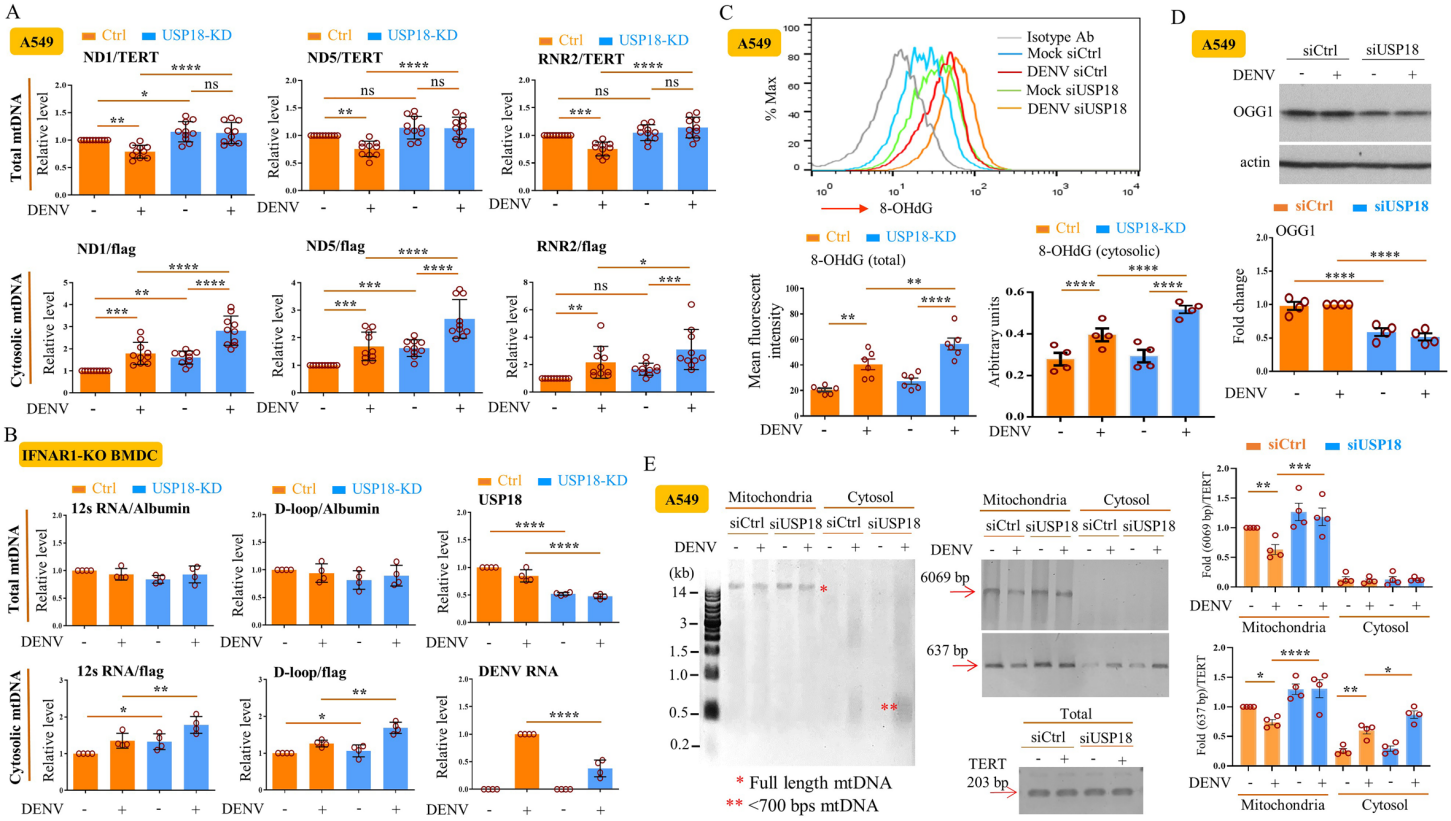
USP18 regulated mtDNA release, oxidation and fragmentation. A549 cells (2 × 10^5^/ml) treated with siCtrl or siUSP18 via lipofectamine 3000 were infected with mock or DENV (MOI = 0.5) for 24 h. Both total and cytosolic DNA were extracted from collected cells according to the Materials and Methods and quantified using qPCR with specific primers to measure mtDNA levels. The total mtDNA content was normalized to nuclear DNA TERT, and the relative abundance of mtDNA in cytosolic fraction was normalized with exogenously added plasmid encoding the FLAG gene (PCR3.1-flag) as described in the Materials and Methods (**A**). BMDCs were prepared from *ifnar1*-KO mice as described in the Materials and Methods and cells (1 × 10^6^/ml) were treated similarly to (A) and mtDNA release was measured (**B**). A549 cells (2 × 10^5^/ml) treated with siCtrl or siUSP18 via lipofectamine 3000 were infected by DENV (MOI = 0.5) for 24 h and the expression of 8OHdG was measured by intracellular immunostaining with anti-8OHdG Abs (1:500) and then analyzed by flow cytometry (**C**, upper and left lower). In addition, the 8OHdG levels in the cytosolic fractions were determined by ELISA (C, right lower). A549 cells were treated similarly to (**C**), collected and the expression of OGG1 in total cell lysates was determined by Western blotting (**D**). A549 cells (2 × 10^5^/ml) were infected by DENV (MOI = 0.5) for 24 h and DNA was prepared from total, mitochondrial, or cytosolic fractions of treated cells as described in the Materials and Methods and run in agarose gels and analyzed by staining with Midori Green Advance Safe DNA/RNA staining kit (**E**, left). The signals of 6069 bp and 637 bp fragments of mtDNA and TERT in individual fractions were amplified by PCR using designated primers (Table 1), analyzed in gels, and followed by ethidium bromide staining (E, right). Statistical analysis was done using two-way ANOVA with Holm-Sidak’s multiple comparisons (**A**–**E**) to compare differences among different treatments. *P < 0.05; **P < 0.01; ***P < 0.001 and ****P < 0.0001. Original gels are presented in Supplementary Fig. 11.

The mitochondrial localization of USP18 suggests that USP18 may exert its effects by regulating the mitochondrial machinery in DENV infection. We focused on studying DENV-induced mtDNA release which activated TLR9^13^. As shown in Fig. 3A and Supplementary Fig. 2, DENV infection reduced total mtDNA levels but increased cytosolic mtDNA levels in A549 cells and HepG2 cells, which were compatible with our observations in DENV-infected hDCs^13^. Notably, USP18 deficiency further enhanced the DENV-induced mtDNA release to the cytosol. The USP18-mediated effects on DENV-induced mtDNA release to the cytosol remained when IFNAR1 was deleted (Fig. 3B). Moreover, USP18 deficiency significantly enhanced DENV infection-induced oxidization of total and cytosolic mtDNA, as determined by measuring the 8-OHdG intensity (Fig. 3C). This conclusion was supported by studies using confocal microscopy demonstrating the colocalization of 8-OHdG with mtDNA and cytosolic DNA (Supplementary Fig. 3). Interestingly, the results in Supplementary Fig. 3 also suggest that siRNA mediated silencing of USP18 notably increased 8-OHdG signal in nuclear DNA in DENV-infected cells. We used an alternative approach to analyze mtDNA oxidation, namely, employing formamidopyrimidine DNA glycosylase (Fpg)-sensitive real-time PCR analysis^15^. As treatment of mtDNA with Fpg removes oxidized purines from the DNA and creates single-strand breaks that block PCR amplification of these sites, the difference in the qPCR amplification between Fpg-treated and Fpg-untreated DNA reflects the presence of oxidative base damage and the percentage of intact DNA; recognition and cleavage by Fpg results in a decrease in the percentage and suggests an increase in the number of sequences harboring oxidized base products^15, 16^. By this approach, we confirmed the enhancement of DENV infection-mediated mtDNA oxidation by USP18 deficiency (Supplementary Fig. 4). The mechanism was likely due to the suppression of OGG1, an enzyme that recognizes and removes 8-OHdG from DNA, in response to USP18 deficiency (Fig. 3D). Because full-length mtDNA cannot be released from mitochondria through mitochondrial pores, we showed that DENV infection increased fragmented mtDNA (size less than 700 bp) in the cytosol and the effect was further enhanced under USP18 deficiency (Fig. 3E and Supplementary Fig. 5).

### Mechanisms underlying the USP18-regulated release of mtDNA

**Figure 4:**
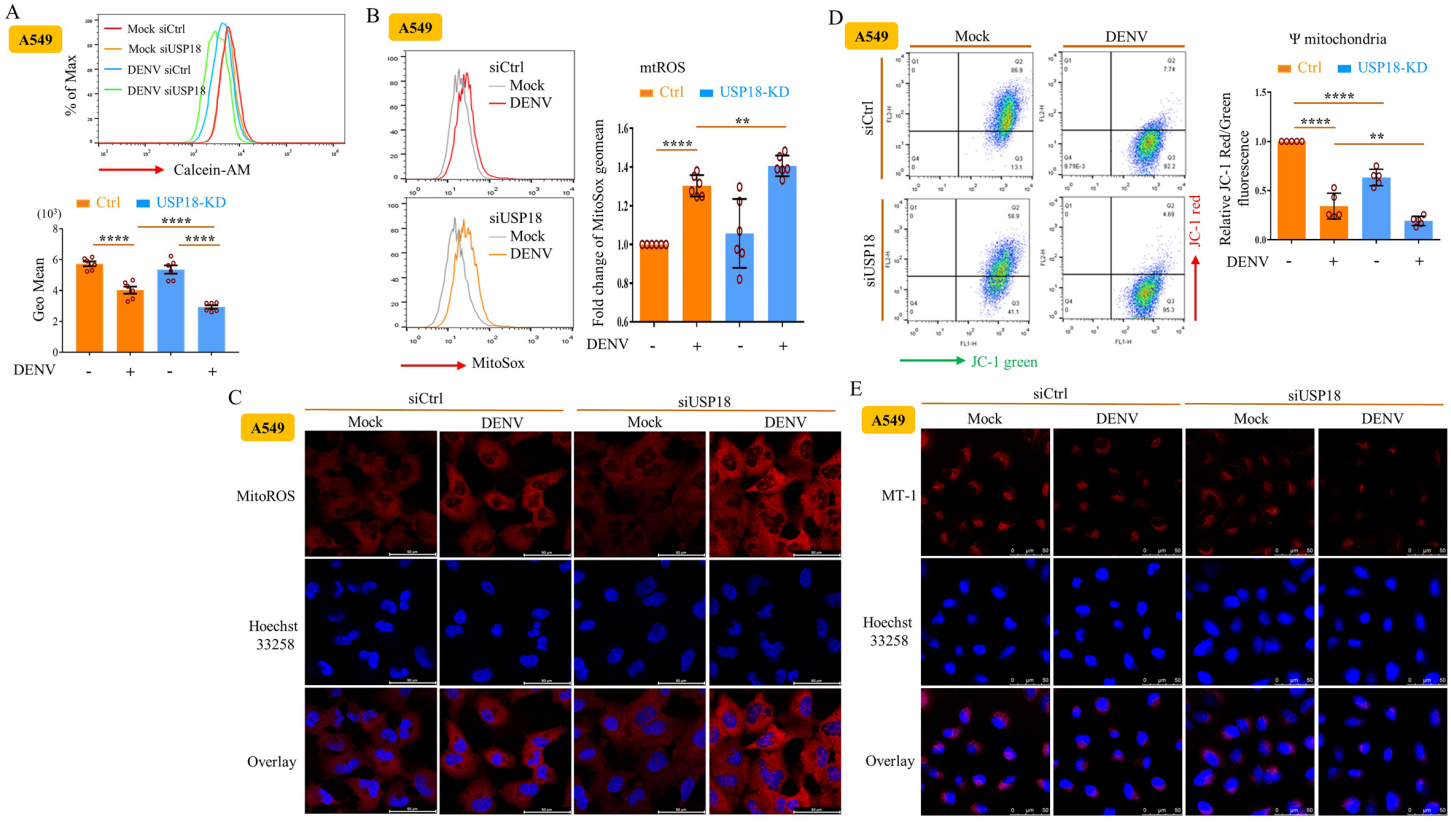
USP18 regulated mtROS production, mPTP opening, and mitochondrial membrane potential changes in DENV infection. A549 cells (2 × 10^5^/ml) treated with siCtrl or siUSP18 were infected with mock or DENV (MOI = 0.5) for 24 h. In the calcein-quenching assay, the treated cells were mixed with 10 nM calcein and 400 μM CoCl_2_, and the fluorescence intensity was determined by flow cytometry (**A**). A549 cells (2 × 10^5^/ml) were infected with mock or DENV (MOI = 0.5) for 24 h, and then 5 μM MitoSOX^TM^Re were added into the culture. After incubation for 0.5 h, the intensity of MitoSOX fluorescence observed via flow cytometry was measured and used as an indicator of mitochondrial ROS levels (**B**). A549 cells treated as described in (**B**) were stained with 1 μg/ml Hoechst 33,258 solution for cell nuclei indicator and MitoSOX Red fluorescence dye for MitoROS and examined with a confocal laser-scanning microscope as described in the Materials and Methods (**C**). Treated A549 cells were stained with JC-1 red/green dye and analyzed by flow cytometry to measure mitochondrial membrane potential as described in the Materials and Methods (**D**). Similar to (**C**), the intensity of mitochondrial membrane potential was analyzed by confocal microscopy after cell staining with MT-1 dye and Hoechst 33,258 (**E**). More than 3 independent experiments were carried out and analyzed for each condition. Statistical analysis was done using two-way ANOVA with Holm-Sidak’s multiple comparisons (**A**, **B** and **D**) to compare differences among different treatments. *P < 0.05; **P < 0.01; ***P < 0.001 and ****P < 0.0001.

**Figure 5:**
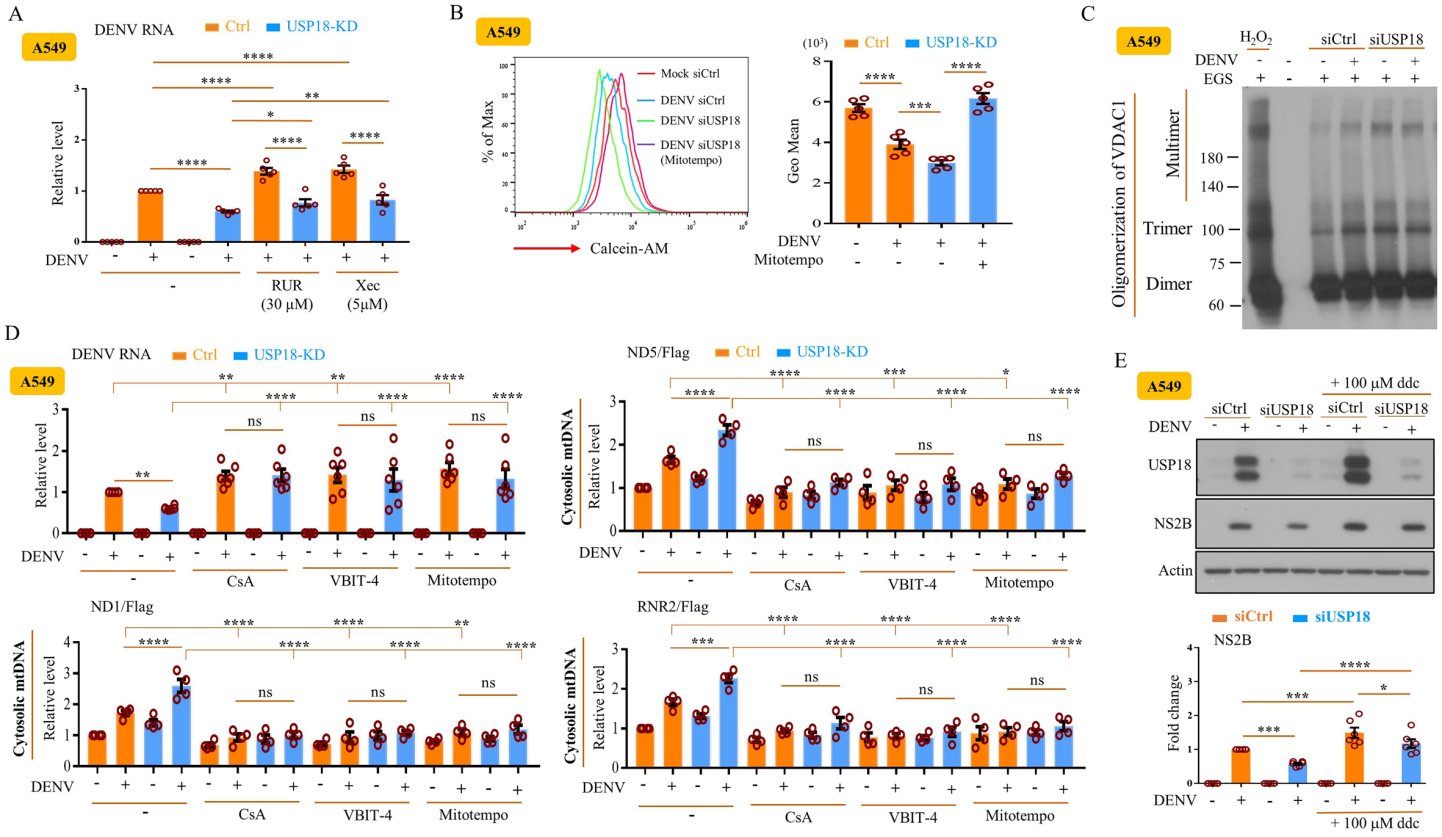
Mechanisms of USP18-regulated mtDNA release examined by specific inhibitors. A549 cells (2 × 10^5^/ml) delivered with siCtrl or siUSP18 were treated with indicated concentrations of RUR and Xec for 2 h and then infected with mock or DENV (MOI = 0.5) for 24 h. The levels of DENV RNA were measured (**A**). A549 cells (2 × 10^5^/ml) delivered with siCtrl or siUSP18 were treated with Mitotempo (100 μM) for 2 h and then infected by DENV (MOI = 0.5) for 24 h. The collected cells were mixed with 10 nM calcein and 400 μM CoCl_2_ in calcein-quenching assays, and the fluorescence intensity was determined by flow cytometry to measure DENV-induced opening of mPTP (**B**). A549 cells (2 × 10^5^/ml) delivered with siCtrl or siUSP18 were infected with mock or DENV (MOI = 0.5) for 24 h. The total cell lysates were incubated with 200 μM EGS at 30 °C for 15 min to stabilize the VDAC oligomer and then examined by Western blot (**C**). H_2_O_2_ (1 mM) treatment for 6 h served as a positive control. A549 cells (2 × 10^5^/ml) delivered with siCtrl or siUSP18 were treated or not with various chemical compounds, including CsA (5 μM), VBIT-4 (10 μM), and Mitotempo (100 μM) for 2 h and then infected by DENV (MOI = 1) for 24 h. The DENV RNA and mtDNA levels were determined. A549 cells (5 × 10^4^/ml) were treated or not with 100 μM ddc for 7 days to deplete mtDNA. Cells were passaged every 2–3 days. The mtDNA-depleted cells (2 × 10^5^/ml) delivered with siCtrl or siUSP18 were infected with mock or DENV (MOI = 0.5) for 24 h. After collecting total cell lysates, the expression of USP18 and DENV NS2B was determined by Western blotting (**E**). More than 3 independent experiments were carried out and analyzed for each condition. Statistical analysis was done using two-way ANOVA with Holm-Sidak’s multiple comparisons (**A**, **B**, **D**, and **E**) to compare differences among different treatments. *P < 0.05; **P < 0.01; ***P < 0.001 and ****P < 0.0001. Original gels are presented in Supplementary Fig. 12.

We subsequently investigated the mechanisms responsible for the effects of USP18 deficiency on mtDNA release. Using calcein-quenching assays to measure mPTP opening, we showed that USP18 deficiency significantly enhanced DENV infection-induced mPTP opening, a reflection of reduced fluorescence signal (Fig. 4A). In addition, USP18 deficiency further increased mitochondrial ROS (mtROS) production compared to DENV infection alone by both flow cytometry (Fig. 4B) and confocal microscopy (Fig. 4C) analysis. USP18 deficiency also enhanced DENV infection-mediated decrease of the mitochondrial membrane potential (Fig. 4D,E) given that the mitochondrial membrane potential reduced by siUSP18 treatment. These findings were also supported by the results examining BMDCs from IFNAR1-KO mice (Supplementary Fig. 6A–C). We then used different specific inhibitors to confirm these events. Treatment with the inositol 1,4,5-trisphosphate receptor (IP3R) antagonist xestospongin C (XeC) and the mitochondrial calcium uniporter (MCU) inhibitor ruthenium red (RuR) to block the mobilization of calcium from the endoplasmic reticulum into mitochondria increased DENV RNA levels and significantly reversed the USP18 deficiency-mediated inhibition of DENV replication (Fig. 5A). These results were further supported by the finding that RuR treatment inhibited the USP18-mediated opening of mPTP (Supplementary Fig. 7A) and the release of mtDNA to the cytosol (Supplementary Fig. 7B). Furthermore, treatment with the ROS inhibitor Mitotempo efficiently inhibited the USP18 deficiency-mediated opening of mPTP (Fig. 5B). Although we observed an increase in voltage-dependent anion-selective channel 1 (VDAC1) oligomerization in USP18-deficient cells, there were no synergistic effects between USP18 deficiency-induced and DENV infection-induced VDAC1 oligomerization (Fig. 5C). Moreover, inhibition of mtROS production and various mitochondrial pore inhibitors reversed the USP18 deficiency-mediated suppression of DENV replication and mtDNA release to the cytosol (Fig. 5D). Finally, the results demonstrated that the cause of mitochondrial dysfunction with 2′,3′ dideoxycytidine (ddC), a nucleoside analog, abolished the USP18 deficiency-mediated suppression of DENV replication, confirming the significance of mitochondrial machineries in USP18-mediated effects (Fig. 5E).

### Overexpression of USP18 enhanced DENV replication and inhibited mtDNA release

**Figure 6:**
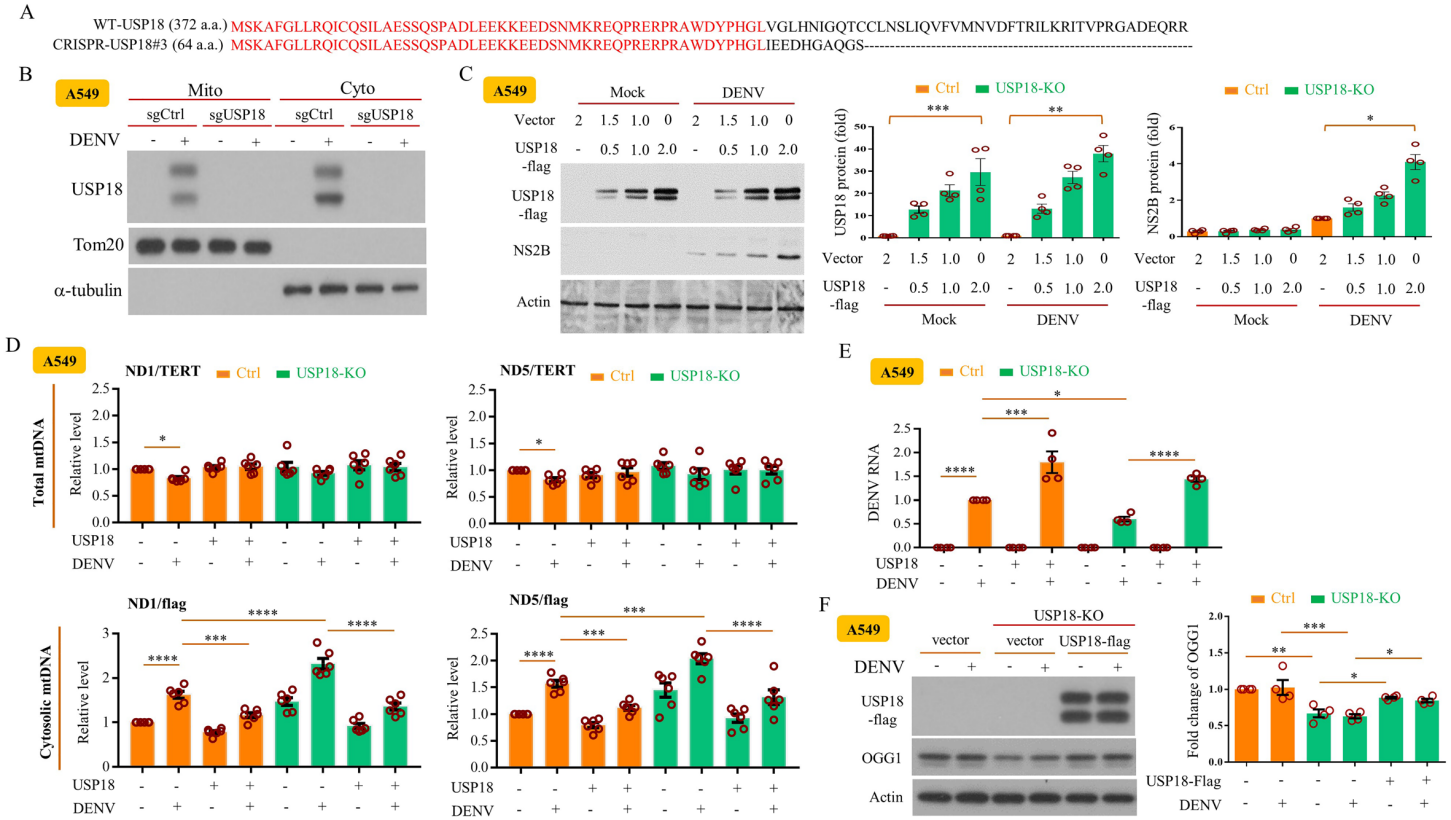
Overexpression of USP18 increased DENV replication in USP18-KO cells. A549 cells were treated with puromycin-selectable lenti-CRISPR vector to deliver control sgRNAs or USP18 sgRNAs to generate USP18-knockout (KO) clone as described in the Materials and Methods. The amino acid sequences of the resulting products were shown (**A**). Expression of USP18, Tom20 and α-tubulin in cytosolic and mitochondrial fractions in wild-type and *usp18*-KO cells was determined by Western blotting (**B**). *usp18*-KO A549 cells (2 × 10^5^/ml) were transfected with different indicated doses of *usp18-flag* plasmid by lipofectamine 3000 (3 μl/ml) to overexpress USP18 and then infected or not with DENV (MOI = 0.5) for 24 h. Expression of USP18 and NS2B was determined and analyzed (**C**). In the wild-type and *usp18*-KO A549 cells (2 × 10^5^/ml) with or without overexpressing USP18, both total and cytosolic fractions were prepared, and mtDNA levels were measured as described in above figures (**D**). The wild-type and USP18-KO A549 cells were transfected with USP18 or control plasmid by lipofectamine 3000. The treated cells were infected by mock or DENV (MOI = 1) for 24 h and the DENV RNA was measured (**E**). The wild-type or USP18-KO A549 cells (2 × 10^5^/ml) were transfected with *USP18-flag* plasmid (2 μg) or control empty vector and then infected by mock or DENV (MOI = 0.5) for 24 h and the expression of USP18 and OGG1 in the whole cell lysates was determined by Western blotting (**F**). Statistical analysis was done using one-way ANOVA Bonferroni (**C**) and two-way ANOVA with Holm-Sidak’s multiple comparisons (**D**–**F**) to compare differences among different treatments. Original gels are presented in Supplementary Fig. 13.

**Figure 7:**
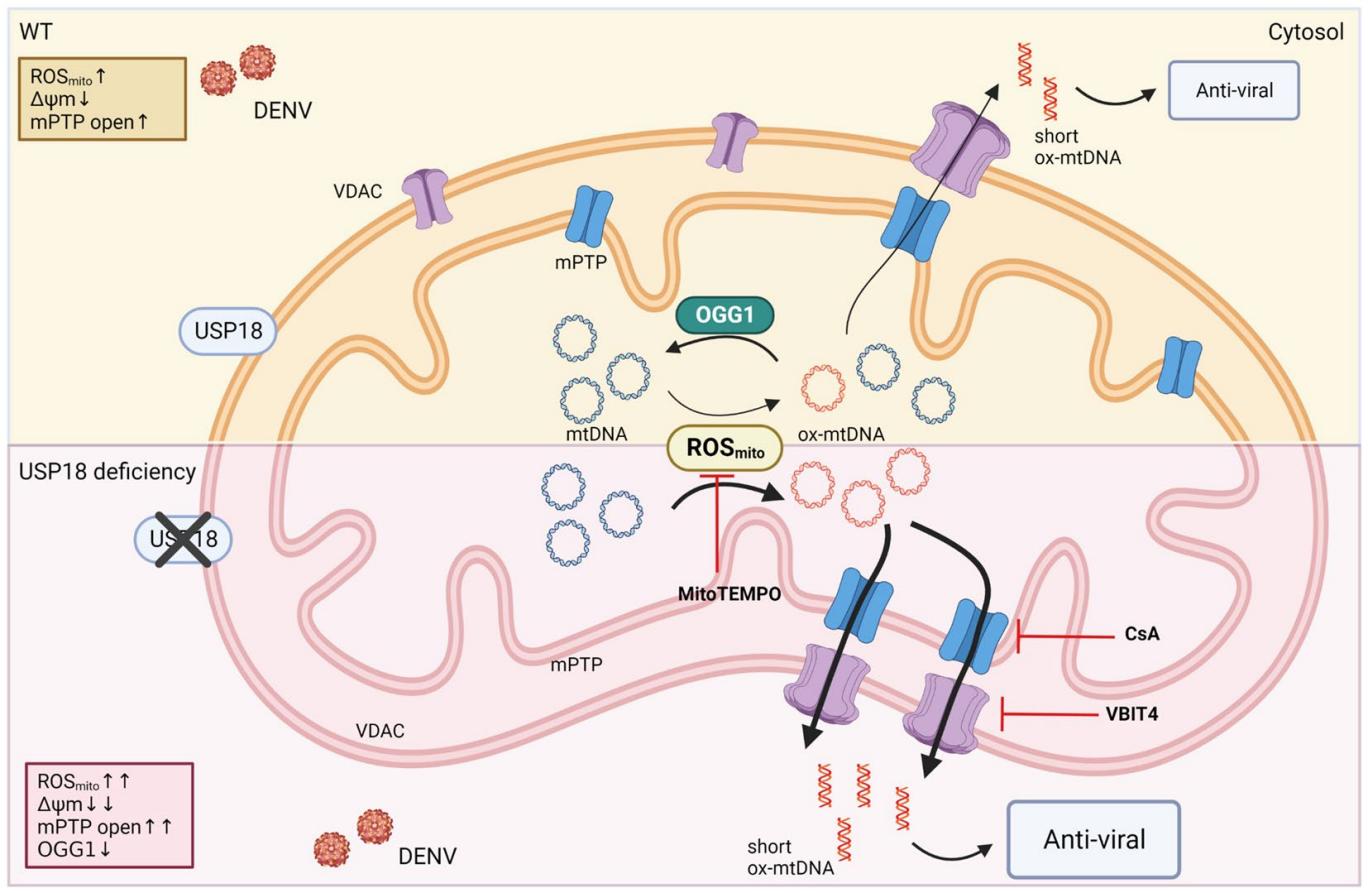
Schematic illustrates how USP18 deficiency regulated mtDNA release in DENV infection. After DENV infection, sequential events occurred in the mitochondria, and some events were enhanced in response to USP18 deficiency. USP18 deficiency resulted in several events, including the enhancement of production of mtROS, calcium mobilization to mitochondria, suppression of OGG1 expression, generation of oxidized mtDNA, formation of mtDNA fragments and opening of mPTP-VDAC pores in DENV infection. These events resulted in the release of oxidized and fragmented mtDNA from mitochondria to the cytosol to activate the downstream signaling pathways. WT, wild-type; ROS, reactive oxygen species; mPTP, mitochondrial permeability transition pore; mtDNA, mitochondrial DNA; OGG1, 8-oxoguanine DNA glycosylase 1; CsA, cyclosporin A; VBIT-4, a voltage-dependent anion channel (VDAC) oligomerization inhibitor. The figure was generated using BioRender.

To further address the mechanisms by which USP18 regulated DENV replication, we generated *usp18*-knockout (KO) A549 cells with the CRISPR technique, which eliminated USP18 in both the cytosol and mitochondrial fractions Fig. 6A,B. Transfection and induced overexpression of USP18-flag upregulated DENV NS2B expression in *usp18*-KO cells in a dose-dependent manner (Fig. 6C). While *usp18*-KO increased the release of mtDNA to the cytosol, overexpression of USP18 effectively inhibited DENV infection-induced mtDNA release (Fig. 6D). Concurrently, overexpression of USP18 enhanced DENV RNA levels (Fig. 6E) and OGG1 expression (Fig. 6F). Furthermore, while *usp18*-KO enhanced mPTP opening in DENV-infected cells, overexpression of USP18 inhibited mPTP opening (Supplementary Fig. 8). The schematic shown in Fig. 7 illustrates how USP18 deficiency regulated mtDNA release to the cytosol in DENV infection.

## Discussion

Reduced virus production in different organs was consistently observed in USP18-deficient or selective USP18 isopeptidase-deficient mice compared to wild-type mice^8, 17, 18^. The increased responsiveness to IFN-I signaling thus has been generally used to explain the effects of USP18 deficiency^17–22^. However, other evidence suggests the existence of wider ranges of USP18 activities beyond its anti-IFN effects. First, USP18 deficiency impairs antiviral immune responses during virus infection^8^. Second, in addition to interfering with the interaction between IFNAR and JAK to block IFN-mediated downstream effects, USP18 can interact with mitochondrial antiviral signaling (MAVS) and the tripartite motif (TRIM) to regulate virus production^11^. Third, USP18 deficiency resulted in a nearly 100-fold reduction in IFN-α2a-mediated downstream effects, but there was only a threefold increase in IFN-α2a-mediated antiviral activity^23^. Fourth, USP18 could promote bacterial replication, and USP18 deficiency inhibited bacterial production in many organs and increased animal survival; these mechanisms were mediated via the suppression of antibacterial TNF signaling^24^. Fifth, depending on the models examined, USP18 can play advantageous^25–27^ or disadvantageous^28, 29^ roles in disease pathogenesis in different animal studies. Finally, in a model of hepatitis C virus infection, Laidlaw et al.^30^ showed that TNF preserved antiviral effects independently of IFNs. Altogether, these results suggest that the effects of USP18 may not completely rely on attenuating the anti-IFN effects. Through different approaches, including the use of JAKs inhibitor, IFN neutralizing Abs, and anti-IFNAR Abs, we showed that USP18 could partly regulate DENV replication in an IFN-unassociated manner. This claim was further supported by studies examining BMDCs from IFNAR1-KO mice and STAT1-KO mice.

mtDNA resembles bacterial nucleic acids and serves as an important damage-associated molecular pattern (DAMP) that is recognized by nucleic acid sensors^31^. Several mechanisms such as changes of mitochondria permeability transition, altered mitophagy and mitochondrial dynamics occurring in conditions such as infection, cell death and inflammation are likely associated with mtDNA escape to the cytosol or the extracellular compartments^32–34^. As 16.3 kb mtDNA is compacted into a nucleoid, it cannot pass through mPTP that have diameters of 2–3 nm^35, 36^. Studies on isolated mitochondria or liposomes revealed that an opened mPTP can only allow the transport of fragmented mtDNA that is less than 700 bp in size and in a single or double helix conformation or linear form^35^. The exact composition of the mitochondrial pores remains unclear; however, some pores responsible for extrusion of mtDNA have been identified that include at least oligomerize pores of N-terminal cleavage product of gasdermin D, BAK/BAX pores, VDAC oligomers-forming pores and the non-specific mPTP^37–43^. In addition to via mitochondrial pores, mtDNA can also be released to the cytosol through the formation of mitochondrial‐derived vesicles^44, 45^. To trigger mtDNA fragmentation and facilitate mtDNA release, oxidation process of mtDNA appeared to be a critical step^32, 41^ where cytidine monophosphate kinase 2 (CMPK2) played roles^14, 46^. Given that many events could potentially be involved in inducing or exaggerating mtDNA oxidation, in the present study examining DENV infection, we demonstrated that the activation by Ca2 + influx, the generation of mtROS, the regulation of OGG1 expression, the oxidization and fragmentation of mtDNA and the opening of the nonspecific mPTP were participating in USP18-regulated mtDNA release to the cytosol. The conclusion was supported by a series of experiments investigating the effects of different specific inhibitors of these events.

There is evidence suggesting that USP18 can interact with MAVS and that this interaction promotes MAVS aggregation, resulting in enhancement of antiviral activity^11^. However, the interaction with MAVS could not explain the observed effects of USP18 in this study, where USP18 deficiency enhanced the release of mtDNA to the cytosol and suppressed viral replication. Recent studies demonstrated that the fragmentation of mtDNA appeared to be upstream of RIG-I–MAVS system activation, and mtRNA serves as a retrograde second messenger of mtDNA stress to stimulate RIG-I–MAVS signaling^47, 48^. This study suggests that the release of mtDNA may occur upstream of MAVS signaling. A recently published report demonstrating that oxidized mtDNA could induce gasdermin D oligomerization and pore formation to facilitate extracellular mtDNA release^49^ suggests possible feedback mechanisms from released oxidized mtDNA to regulate mitochondrial machineries.

### Limitations of the study

The evident limitation of this study is the lack of animal data to support the significance of the observed findings in our cellular system. Nevertheless, differences were observed in USP18-deficient animals that were infected with different viruses via different infection routes. While USP18-deficient mice exhibited better protection from intracerebral challenge with lethal lymphocytic choriomeningitis virus (LCMV) or VSV infection than wild-type mice^17^, intravenous administration of VSV led to virus-mediated paralysis and early death in USP18-deleted mice^8^. In addition, there were no images showing the movement of mtDNA and the opening of mPTP, which was regulated by USP18. Which molecule is targeted by and interacts with USP18 to mediate its effects is also unclear in this study. We are currently planning more studies to address these questions.

## Materials and methods

### Cell culture

Human lung epithelial A549 cells and liver HepG2 cells (Bioresource Collection and Research Center, Taiwan) were maintained in F12K medium and MEM, respectively, supplemented with 10% fetal bovine serum (FBS) in a humidified atmosphere containing 5% CO_2_ at 37 °C. Liver Huh7 cells (Gift from Dr. Cai-yuan Xie, Tri-Service General Hospital) were maintained in DMEM, respectively, supplemented with 10% fetal bovine serum (FBS) in a humidified atmosphere containing 5% CO_2_ at 37 °C.

### Reagents

Antibodies (Abs) against murine USP18 (A16739), and OGG1 (A4997) were purchased from ABclonal (Woburn, MA, USA). Anti-human USP18 Abs (#4813) were purchased from Cell Signaling Technology (Beverly, MA, USA). Anti-ISG15 Abs (PA5-17461) were obtained from Invitrogen (Carlsbad, CA, USA). Anti-DENV NS2B (GTX124246), anti-DENV NS3 (GTX629477), β-actin (GTX109639), anti-Flag (GTX115043) and goat IgG isotype control Abs (GTX35039) were purchased from GeneTex Inc. (Irvine, CA, USA). Anti-STAT1 Abs (SC-592) were purchased from Santa Cruz Biotechnology (Dallas, TX, USA). Anti-phosphorylated STAT1 Abs (2825-1) were purchased from Epitomics (Burlingame, CA, USA). Anti-Tom20 Abs (ab186734) were purchased from Abcam (Cambridge, UK). Anti-α-tubulin Abs (NB100-690) were purchased from Novus (Briarwood Avenue, CO, USA). Anti-8-OHdG Abs (AB5830) were from Millipore (Burlington, MA, USA). Anti-8-OHdG Abs for ELISA (SKT-120-96S) were purchased from StressMarq Biosciences Inc (Victoria, BC V8N 4G0, Canada). Donkey anti-Rabbit IgG Abs (H + L), Alexa Fluor 488 (A-21206), Donkey anti-Goat IgG (H + L), Alexa Fluor 594 (A-11058), MitoSOX (M36008), JC-1 (M34152), ProLong Diamond Antifade Mountant (P36970), Lipofectamine 3000 (L3000001) and Calcein AM (C1430) were purchased from Thermo Fisher Scientific (Waltham, MA, USA). Anti-IFN-α Abs (21100-1) (used at 5 ug/ml), Anti-IFN-α receptor 1 (21370-1) (used at 5 ug/ml), recombinant human IFN‐α (11100-1) (used at 100 U/ml), recombinant mouse IFN‐α (12100-1) (used at 100 U/ml), and recombinant human IFN‐β (11514-1) (used at 50 U/ml) were purchanced from PBL Assay Science (Piscataway, NJ, USA). MitoView Fix 640 (70,082), used similar to mitotracker, was purchanced from Biotium, Inc (Landing Parkway Fremont, CA, USA). Human GMCSF (215-GM) (used at 800 U/ml), human recombinant IL-4 (204-IL) (used at 500 U/ml), IFN‐γ (285-IF) (used at 10 ng/ml), and TNF-α (210-TA) (used at 40 ng/ml) were purchanced from R&D, Inc (New York, NY, USA). Mouse GMCSF (31503) (used at 20 ng/ml) and recombinant human IFN‐λ1 (300-02L) (used at 40 ng/ml) were purchanced from PeproTech, Inc (Rehovot, Israel). VBIT-4 (HY129122) was purchased from MedChemExpress (Monmouth Junction, NJ, USA). Ruxolitinib (tlrl-rux) was purchased from Invivogen (San Diego, CA, USA). Human CD14 + microbead (130-050-201) was purchased from Miltenyi Biotec (Bergisch Gladbach, North Rhine-Westphalia, Germany). Xestospongin C (64,950) was purchased from Cayman Chemical (Michigan 48,108 USA). Fpg enzyme (M0240S) was purchased from New England Biolabs (Ipswich, MA, USA). Midori Green Advance Safe DNA/RNA stain (MG04) was purchased from NIPPON Genetics Europe (Mariaweilerstraße Düren, Germany). PfuUltra II Fusion High-fidelity DNA Polymerase (600,670) was purchased from Agilent Technologies (Santa Clara, CA, United States). MT-1 MitoMP Detection Kit (MT13) was purchased from Dojindo (Kumamoto, Japan). Unless otherwise specified, all other reagents were purchased from Sigma Aldrich.

### DENV preparation and infection

Preparation of DENV has been previously described^50^. The DENV2 strain New Guinea C was propagated in C6/36 mosquito cells in RPMI medium containing 5% heat-inactivated FBS and maintained at 28 °C for 7 days. Preparation of mock conditioned medium was performed using the same procedures, except buffered saline was substituted for virus inoculation. Unless specified, DENV at multiplicity of infection (MOI) 0.5 or 1 was consistently used to infect cells.

### Preparation of human monocyte-derived dendritic cells and mouse bone marrow-derived dendritic cells (BMDCs)

Human monocyte-derived dendritic cells (hDCs) were prepared as described in our previous report^13^. After collection of peripheral blood mononuclear cells (PBMCs) from buffy coat (purchased from Blood Donation Center, Taipei, Taiwan), the CD14^+^ monocytes were isolated using a magnetic-activated cell isolation column (Miltenyi Biotec). The purified monocytes were cultured in RPMI 1640 medium containing 10% FBS, 800 U/ml granulocyte macrophage-colony stimulating factor (GM-CSF) and 500 U/ml interleukin (IL)-4 at a cell density of 1 × 10^6^ cells/ml. The culture medium was replaced every other day with fresh medium containing GM-CSF and IL-4, and hDCs with a purity greater than 95% after 5–7 days of culture were used in the experiments^50^. The preparation of mouse BMDCs was performed according to our previous report^13^. In brief, male C57BL/6 mice (6–12 weeks) were purchased from the National Laboratory Animal Breeding and Research Center (Taipei, Taiwan). The *Ifnar1-/-* mice were from Dr. Guann-Yi Yu (National Health Research Institute (NHRI), Taiwan) and *Stat1*-/- mice were provided by Dr. Chien-Kuo Lee (National Taiwan University, Taiwan). All the animal studies were conducted in accordance with a protocol approved by the Institutional Animal Care and Use Committee of the National Health Research Institute (NHRI; NHRI-IACUC-107159-A-S01) and were also carried out in compliance with the ARRIVE guidelines (https://arriveguidelines.org). Bone marrow was flushed from the tibias and femurs of mouse hind legs using a needle syringe loaded with DMEM. After washing and filtering through a 40-mm nylon cell strainer, bone marrow cells were cultured in RPMI with 10 ng/ml of mGM-CSF (PeproTech Inc., New Jersey, USA) for 6 days with the medium refreshed every 2–3 days for BMDCs.

### Determination of virus titers

To determine virus titers, the culture supernatants were harvested for plaque-forming assays^50^. Various virus dilutions were added to 80% confluent baby hamster kidney (BHK-21) cells and incubated at 37 °C for 1 h. After adsorption, cells were washed and overlaid with 1% agarose (SeaPlaque; FMC BioProducts) containing RPMI 1640 and 1% FCS. After incubation for 7 days, cells were fixed with 10% formaldehyde and stained with 0.5% crystal violet. The numbers of plaques were counted, and the results were shown as plaque forming unit (PFU) per milliliter.

### Western blotting

Enhanced chemiluminescence Western blotting (Amersham, GE Healthcare Life Science, Uppsala, Sweden) was performed to study protein levels. Briefly, proteins were separated on a SDS-PAGE gel and transferred to a nitrocellulose membrane. For immunoblotting, the nitrocellulose membrane was incubated with TBS-T containing 5% nonfat milk for 1 h at room temperature. Then blotted with Abs against a specific protein for 2 h at room temperature or overnight at 4 °C. After washing, the membrane was incubated with secondary Abs conjugated to horseradish peroxidase for 1 h at room temperature. The membrane was then incubated with a substrate and exposed to X-ray film.

### siRNA transfection

The sequence for USP18 siRNA were GGAUCUACGGAGUCUUCUA (Homo sapiens) and GUGGAUGGAAAGUGGUUCU (Mus musculus). Primary cells were collected and resuspended at 1 × 10^7^ ml in modified Eagle's minimum essential medium (opti‐MEM, Invitrogen) containing 300 nM designated siRNA (Stealth RNAi™ siRNA, Invitrogen). Electroporation was performed using a BTX electroporator (San Diego, CA) with a profile of one pulse at 300 V for 3 ms. The cells (2 × 10^6^) were then seeded in culture medium (Invitrogen, Carlsbad, CA, USA) containing 10% FBS for 24 h before subsequent treatment. For cell lines, cells were transfected with 50 nM siRNA by using Lipofectamine 3000 (3 μl/ml, Invitrogen). After transfection for 4 h, the culture medium was replaced with fresh complete medium for further experiments.

### Preparation of cytosolic and mitochondrial fractions

A Mitochondria/Cytosol Fractionation Kit from Abcam (Cambridge, UK) was used to extract mitochondrial and cytosolic fractions as described by the manufacturer. In brief, 2 × 10^7^ cells were resuspended in 0.3 ml of 1X Cytosol Extraction Buffer Mix supplemented with dithiothreitol (DTT) and protease inhibitors. After incubation on ice for 10 min, the cells were homogenized in an ice-cold Dounce tissue homogenizer (150–200 passes with the grinder). The homogenate was centrifuged at 700 × g in a microcentrifuge for 10 min at 4 °C, and the supernatant was then centrifuged at 10,000 × g in a microcentrifuge for 30 min at 4 °C. Then, the supernatant was collected (cytosolic fraction), and the pellet (intact mitochondria) was resuspended in 50 μl of the Mitochondrial Extraction Buffer Mix supplemented with DTT and protease inhibitors (mitochondrial fraction).

### Measurement of mtROS levels

For mtROS level measurement, the cells were incubated with 5 μM MitoSOX^TM^Red (Invitrogen) in cultured medium for 30 min at 37 °C. After washing with PBS, the cells were suspended with trypsin–EDTA and were analyzed with flow cytometry^13^.

### USP18-knockout A549 cells

USP18 sgRNA was designed and cloned into a CRISPR‒Cas9 system by the National RNAi Core Facility (RNA technology platform and gene manipulation core, Academia Sinica, Taiwan). The sequence for USP18 sgRNA were GTTCCCCTTATAGGCCTGGT (Homo sapiens). A549 cells were transfected with the plasmid carrying USP18 sgRNA for 48 h. Then, puromycin (10 μg/ml) was added to the culture medium, and the cells were incubated for 7 days; the medium was regularly replaced to eliminate untransfected cells. Subsequently, a single CRISPR‒Cas9 USP18-knockout (KO) A549 clone was selected, picked and transferred to 12-well plates for further culture. Successful KO was confirmed by Western blotting and DNA sequencing.

### Overexpression of USP18 in A549 cells

A construct of human USP18 retrieved from the A549 cDNA library was cloned into pcDNA3.1 + and pcDNA3.1 + /C − (K) − DYK (GenScript Inc. Piscataway, NJ). The accuracy of the subcloning was confirmed by sequencing. For overexpression of USP18 in A549 cells, the indicated concentrations of human USP18 were transfected into A549 cells with Lipofectamine 3000 reagent according to the manufacturer’s instructions.

### Quantitative RT‒qPCR

Total RNA was isolated from treated cells with NucleoZOL reagent (Macherey-Nagel, Duren, Germany) according to our previous report^14^. RNA concentrations were measured using a NanoDrop spectrophotometer (ND 1000 V.3.1.0; Thermo Fisher Scientific, Waltham, MA, USA). Reverse transcription was performed with a 20 μl mixture containing 2 μg of total RNA, random hexamers (Invitrogen), and a mixture containing 10X reverse transcription buffer, dNTPs, magnesium chloride, DTT, and Moloney Murine Leukemia Virus Reverse Transcriptase (MMLV RTase, Invitrogen). cDNA was prepared for further evaluation using qPCR. Briefly, 20 ng of cDNA was amplified in a total mixture volume of 20 μL consisting of 1 × KAPA SYBR FAST qPCR Master Mix (KAPA Biosystems, Boston, MA, USA) and the appropriate gene-specific primers, which were added at a final concentration of 200 nM. The primers used are shown in Table 1. The reaction conditions were 40 cycles comprising steps at 95 °C for denaturing and 60 °C for annealing and extension on a LightCycler 480 (Roche). The changes in gene expression induced by DENV infection in the presence or absence of inhibitors or siRNA were calculated with the following formula: fold change = 2^−Δ(ΔCt)^, where ΔCt = Ct of target gene − Ct of GAPDH, and Δ(ΔCt) = ΔCt infected − ΔCt mock control.

**Table 1:** Summary of primer sequences used in the study.

	Accession number	Forward	Reverse
Gene name (*Homo sapiens*)			
USP18	NM_017414.3	CCTGAGGCAAATCTGTCAGTC	CGAACACCTGAATCAAGGAGTTA
GAPDH	NM_001289746.1	AGGTGAAGGTCGGAGTCAAC	CCATGTAGTTGAGGTCAATGAAGG
mtDNA-ND1	KY399206.1	TTCTAATCGCAATGGCATTCCT	AAGGGTTGTAGTAGCCCGTAG
mtDNA-ND5	KY399206.1	TTCATCCCTGTAGCATTGTTCG	GTTGGAATAGGTTGTTAGCGGTA
mtDNA-RNR2	KY399206.1	AAATCTTACCCCGCCTGTTT	GGCAGGTCAATTTCACTGGT
mtDNA (637 bp)	KY399206.1	TTCTAATCGCAATGGCATTCCT	ATGAAGAATAGGGCGAAGGG
mtDNA (6069 bp)	KY399206.1	CTCCTCAAAGCAATACACTG	ATTCCGAAGCCTGGTAGGAT
TERT	NM_198253.3	CTTCCTCTACTCCTCAGGCG	CAAGCAGCTCCAGAA ACAGG
Gene name (*Mus musculus*)			
USP18	NM_011909	GGAACCTGACTAAGGACCAGATC	GAGAGTGTGAGCAGTTTGCTCC
IFN-α	NM_010503.2	AAGGACAGGCAGGACTTTGGATTC	GATCTCGCAGCACAGGGATGG
IFN-β1	NM_010510.2	CAGCTCCAAGAAAGGACGAAC	GGCAGTGTAACTCTTCTGCAT
12S	KY018919.1	ACCGCGGTCATACGATTAAC	CCCAGTTTGGGTCTTAGCTG
D-loop	KY018919.1	GCCCATGACCAACATAACTG	CCTTGACGGCTATGTTGATG
Albumin	NC_000071.6	TGAAACATATGTCCCCAAAGAGTTT	TTCTCCTTCTCTGGAAGTGTGCAGA T
ISG15	NM_015783	CAATG GCCTG GGACC TAAA	CTTCT TCAGT TCTGA CACCG TCAT
*Mx1*	NM_010846.1	GGGGA GGAAA TAGAG AAAAT GAT	GTTTA CAAAG GGCTT GCTTG CT
*Ifit3*	NM_010501.2	AGCCCACACCCAGCTTTT	CAGAGATTCCCGGTTGACCT
*Ifit1*	NM_008331.3	CAAGG CAGGT TTCTG AGGAG	GACCT GGTCA CCATC AGCAT
*Oas1*	NM_001083925.1	GCTGA TGTCA AATCA GCCGT	AGCTT GAAGC TCAGA GACC
Others			
DENV2	KY586699.1	CTCTCAGTGAACTGCCGGAGACC	CGTACCATAGGAGGATGCTAGCCG
pCR3.1-Flag		GAAAAGTGCCACCTGACGC	GCCCCCGATTTAGAGCTTGA

### Extraction of total, cytosolic and mitochondrial DNA

Total and cytosolic DNA extraction was performed according to our previous report^13^. Cells were divided into two equal aliquots. One aliquot, the control for normalization, was used to extract total DNA using a NucleoSpin Tissue kit (Macherey-Nagel, Duren, Germany). The other aliquot was resuspended in 400 ml of buffer containing 150 mM NaCl, 50 mM HEPES pH 7.4, and 25 mg/ml digitonin (EMD Chemicals, Gibbstown, NJ, USA). After rotation end-over-end for 15–20 min at 4 °C, the samples were then centrifuged at 980 × g for 3 min three times to remove cellular debris. The supernatants were collected, transferred to fresh tubes, and spun at 17,000 × g for 10 min to collect lysates (the mitochondrial fraction) and supernatants (the cytosolic fraction). The DNA in the mitochondria, cytosol and total fraction were then isolated by running the sample through a NucleoSpin Tissue column (Macherey-Nagel, Duren, Germany), subsequently eluted with buffer, and analyzed on 1% agarose gel that was stained with Midori Green Advance Safe DNA/RNA stain (NIPPON Genetics Europe, Duren, Germany).

### Determination of mtDNA

To measure the levels of mtDNA, 20 ng of isolated DNA was subjected to qPCR with KAPA SYBR FAST qPCR Master Mix (KAPA Biosystems) with 200 nM mtDNA primers (ND1, ND5, and RNR2 for A549 and HepG2 cells; 12S and D-loop for BMDCs) or nuclear DNA (nDNA) primers (TERT for A549, and HepG2 cells; albumin for BMDCs). To quantify mtDNA in the cytosolic fraction, 20 ng of a purified plasmid encoding the FLAG gene (PCR3.1-flag) was added to the eluted solution according to our previous report^13^. The specific primers for both endogenous mtDNA and the FLAG plasmid were used to measure the relative content of cytosolic mtDNA expression normalized to that of FLAG (the primers for PCR are given in Table 1). The relative mtDNA abundance indicates the relative mtDNA content in DENV-infected cells normalized to that in mock-infected cells.

### Measurement of mtDNA fragments

To amplify 637 or 6069 bp mtDNA fragment and telomerase reverse transcriptase (TERT) from the mitochondrial, cytosolic or total DNA pool of treated cells, we followed the method described by other researchers^40^ with some modifications. In brief, 10 ng of the extracted cytosolic and mitochondrial DNA was used as the template. In addition to the template, the reaction mixture also contained 200 nM of each primer and PfuUltra II Fusion HS DNA Polymerase (Agilent Technologies, Santa Clara, CA, USA). The PCR conditions were as follows: 95 °C for 10 min, melting temperature of 95 °C for 60 s, annealing temperature of 55 °C (for 6069 bp and 637 bp) for 1 min 30 s or 59 °C (for TERT) for 1 min and extension temperature of 72 °C for 30 s (637 bp and TERT) and 3 min (6069 bp). Then, the reaction was run for 24 (637 bp and TERT) or 40 (6069 bp) cycles, followed by incubation at 72 °C for 20 min, and the samples were maintained at 4 °C. The amplified products were analyzed on 1% agarose gels that were stained with ethidium bromide.

### Measurement of mitochondrial membrane potential with JC-1 probe

The fluorescent probe, JC-1, purchased from Life Technologies was used to study mitochondrial membrane potential according to the manufacturer’s instructions and our published report^13^. In brief, cells (2.5 × 10^5^ cells per each condition) were incubated with JC-1 staining solution for 30 min at 37 °C. After washes with PBS, the fluorescence intensity of JC-1 was measured by a flow cytometer (Becton Dickinson) and the results were analyzed with a FlowJo software (Becton Dickinson).

### Immunofluorescence staining

Cells were collected and washed two times with PBS before being fixed with 4% paraformaldehyde on ice for 20 min^13^. The fixed cells were washed with PBS and permeabilized with 0.2% Triton X-100 in PBS for 10 min. Cells were washed again with PBS and blocked with PBS containing 10% BSA for 30 min. Primary Abs were added and incubated with the cells for 2 h at room temperature with occasional mixing. After removing unbound Abs by washing, secondary Abs conjugated with fluorescent dye were added and incubated in the dark for 1 h at room temperature with occasional mixing. Cellular nuclei were counterstained with 1 μg/ml DAPI or Hoechst 33,258 solution. For detecting the colocalization of DNA and mitochondria, MitoView Fix 640 fluorescence dye was used for staining mitochondria. For detecting oxidative DNA and DENV, anti-8-OHdG and anti-NS2B Abs were used. Finally, cells were mounted with mounting reagent (ProLong Diamond Antifade Mountant, Thermo Fisher Scientific) for subsequent confocal microscopy analysis.

### Confocal microscopy analysis

Samples were examined with a Leica TCS SP5II and Stellaris 8 confocal laser-scanning microscope (Leica Microsystems, Wetzlar, Germany) equipped with HCX PL APO 63 × /1.4–0.6 oil and HC PL APO 100x/1.40 oil objective (Leica) in core facilities of NHRI according to our previous publication^13^. Image processing and colocalization analysis were performed with Leica LAS AF and LAS × life science microscope software.

### Determination of 8‐OHdG levels with flow cytometry and ELISA

To determine oxidized DNA levels in the mitochondrial and cytosolic fractions^51^, 500 ng of DNA was added to poly-L-lysine-coated (0.01%) 96-well EIA/RIA plates (Costar, Washington, DC, USA) and incubated at 4 °C overnight. The wells were washed with PBS and blocked with PBS supplemented with 1% BSA for 2 h at room temperature. A horseradish peroxidase-conjugated mouse anti‐human 8‐OHdG Abs (StressMarq Biosciences Inc., Columbia, Canada) that recognizes oxidized nucleotides was added at 0.5 μg/ml and incubated for 2 h at room temperature. After being washed with PBS, the samples were incubated with substrate for 30 min, the reaction was stopped by adding stop solution, and the absorbance was read at 450 nm with an ELISA reader.

For intracellular 8-OHdG detection, cells were washed twice with PBS and detached with trypsin. After fixation with 4% paraformaldehyde, the cells were permeabilized with 0.05% Triton X-100 and then blocked with PBS supplemented with 1% BSA. Goat anti-human 8‐OHdG Abs (Millipore) was added and incubated for 1 h at room temperature. After washing twice with PBS, donkey anti-goat Alexa Fluor™ 488 was added and incubated for 0.5 h at room temperature. After washing twice with PBS, the samples were analyzed with flow cytometry.

### Mitochondrial permeability transition pore (mPTP) opening assay

To analyze mPTP opening by calcein-quenching assay, treated cells were detached from the culture plates by trypsin–EDTA. The cells were mixed with 10 nM calcein and 400 μM CoCl_2_ for 10 min at 37 °C according to the manufacturer’s instructions (Thermo Fisher Scientific, Waltham, MA USA). After washing twice, the fluorescence intensity was determined by flow cytometry. To analyze mPTP opening in BMDCs, we followed the method described by other researchers^52^. BMDCs were mixed with 1 μM calcein-AM and 1 mM CoCl_2_ for 10 min at 37 °C. After washing twice, the fluorescence intensity was determined by flow cytometry.

### Fpg-sensitive qPCR

The measurement of mtDNA oxidative damage with formamidopyrimidine DNA glycosylase (Fpg)-sensitive qPCR analysis was performed according to the report^40^. In brief, 250 ng of purified mtDNA was incubated with 8 units of Fpg in 1X NEBuffer 1 and 100 mg/mL BSA in 50 mL at 37 °C for 1 h. After inactivating the Fpg enzyme by incubation at 60 °C for 5 min, 10 ng of DNA was used for qPCR to detect Fpg-sensitive cleavage sites. Data are calculated as the quotient of signal intensities in Fpg-treated DNA relative to Fpg-untreated DNA and reflect the fraction of intact DNA.

### VDAC1 cross-linking assay

Treated cells were washed with PBS and incubated with the cross-linking reagent EGS (200 μM) for 15 min at 30 °C^40^. The samples were analyzed with SDS‒PAGE, and the membranes were blotted with anti-VDAC1 Abs.

### Statistical analysis

Statistical comparisons were performed using Student’s *t*-test (paired t test for primary cells and unpaired t for cell lines) or one-way analysis of variance (ANOVA). When ANOVA showed significant differences between groups, Bonferroni’s post hoc test was used to determine the specific pairs of groups that significantly differed. For multiple comparisons, two-way ANOVA with Holm-Sidak’s multiple comparisons was applied. A *P* value of < 0.05 was considered to indicate statistical significance. Asterisks indicate values that are significantly different from the relevant control (*P < 0.05, **P < 0.01, ***P < 0.001 and ****P < 0.0001).

### Supplementary Information

Supplementary Figures.

## Data Availability

The data sets used in this study are available from the corresponding author on reasonable request.
