# Supplementary material for: USP18 enhances dengue virus replication by regulating mitochondrial DNA release
Source: Sci Rep. 2023 Nov 17;13:20126. doi: 10.1038/s41598-023-47584-w (PMC10656416; doi:10.1038/s41598-023-47584-w)
Supplement: Supplementary file 1 — Supplementary Figures. [file 41598_2023_47584_MOESM1_ESM.docx]

**Supplementary materials**

USP18 enhances dengue virus replication by regulating mitochondrial DNA release

Jenn-Haung Lai ^a,*^, De-Wei Wu ^a^, Chien-Hsiang Wu ^a^, Li-Feng Hung ^b^, Chuan-Yueh Huang ^b^, Shuk-Man Ka ^c^, Ann Chen ^d^, Ling-Jun Ho ^b,*^

^*^ **Correspondence address**:

Jenn-Haung Lai, MD, PhD. Division of Allergy, Immunology and Rheumatology, Department of Internal Medicine, Chang Gung Memorial Hospital, Tao-Yuan, Taiwan, R.O.C. Tel: 886-2-8792-7135; Fax: 886-2-8792-7136 E-mail: [laiandho@gmail.com](mailto:laiandho@gmail.com) and/or Ling-Jun Ho, PhD, [Institute of Cellular and System Medicine](http://english.nhri.org.tw/inst_system/index.php), National Health Research Institute, Zhunan, Taiwan, ROC. Tel. : +886-2-8791-8382; Fax: +886-2-8791-8382; Email: [lingjunho@nhri.org.tw](mailto:lingjunho@nhri.org.tw)


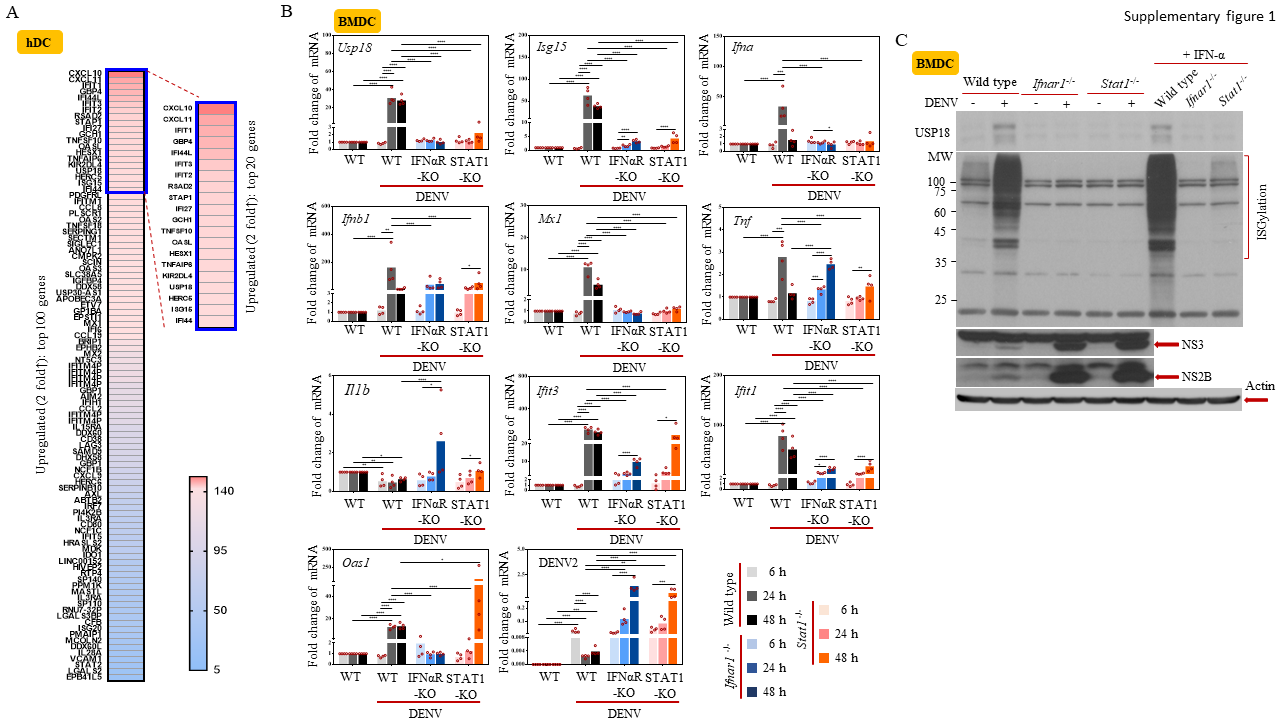


**Supplementary figure 1.** Human dendritic cells (DCs) (5 x 10^6^/ml) were infected with DENV (MOI=5) or mock for 24 h and cellular RNA was collected and analyzed by microarray (A). USP18 was among top 20 of DENV-induced genes. Induction of mRNA or USP18, IFNs and ISGs in *ifnar1*-KO and *stat1*-KO BMDCs (used at 5 x 10^6^/ml) after DENV (MOI=1) or mock infection (B). The DENV-induced expression of USP18, viral NS2B and NS3 glycoproteins, and protein ISGylation in BMDCs from wild-type, *ifnar1*-KO and *stat1*-KO mice (C). More than 3 independent experiments were carried out and analyzed. Statistical analysis was done using one-way ANOVA (B) to compare differences among different treatments. *, P < 0.05; **, P < 0.01; ***, P < 0.001 and ****, P < 0.0001. The original gels in 1C were presented in Supplementary figure 14 (1 and 2).


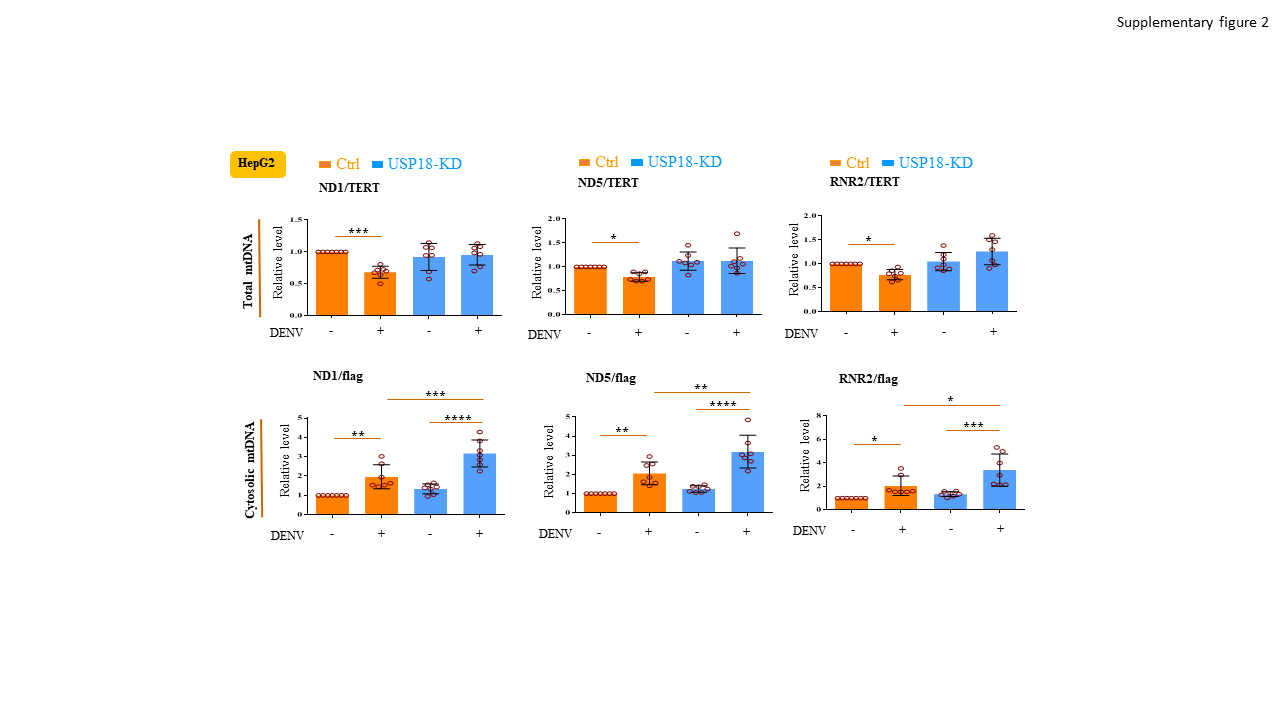


**Supplementary figure 2. USP18 regulated mtDNA release in HepG2 cells.** HepG2 cells (2 x 10^5^/ml) transfected with siCtl or siUSP18 (300 nM) via lipofectamine 3000 were infected with mock or DENV (MOI=0.5) for 24 h were collected, and both total DNA and cytosolic DNA were extracted according to the Materials and Methods and quantified using qPCR with specific primers to measure the mtDNA levels. The relative abundance of mtDNA levels was determined by normalization with the exogenously added 20 ng of a purified plasmid encoding FLAG gene (PCR3.1-flag) as described in experimental procedures. More than 3 independent experiments were carried out and analyzed. Statistical analysis was done using two-way ANOVA with Holm-Sidak to compare differences among different treatments. *, P < 0.05; **, P < 0.01; ***, P < 0.001 and ****, P < 0.0001.


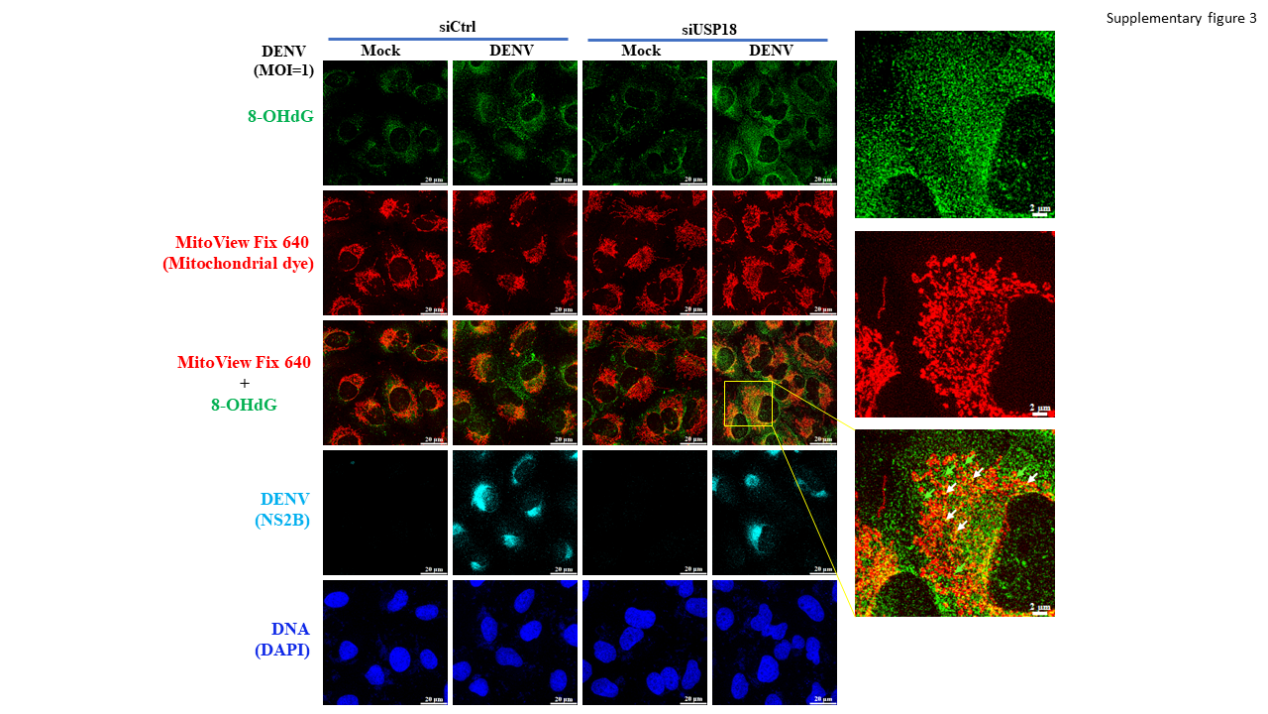


**Supplementary figure 3. USP18 regulated mtDNA oxidation**

A549 cells (2 x 10^5^/ml) treated with siCtrl or siUSP18 were infected with mock or DENV (MOI=1) for 24 h. Treated cells were stained with DAPI, 8OHdG, mitochondria dye MitoView Fix 640 and DENV NS2B and analyzed by confocal microscopy. More than 3 independent experiments were carried out and analyzed. White arrows: 8-OHdG oxidative DNA co-localizing with mitochondria (yellow color). Green arrows: 8-OHdG oxidative DNA in cytosol (green color).


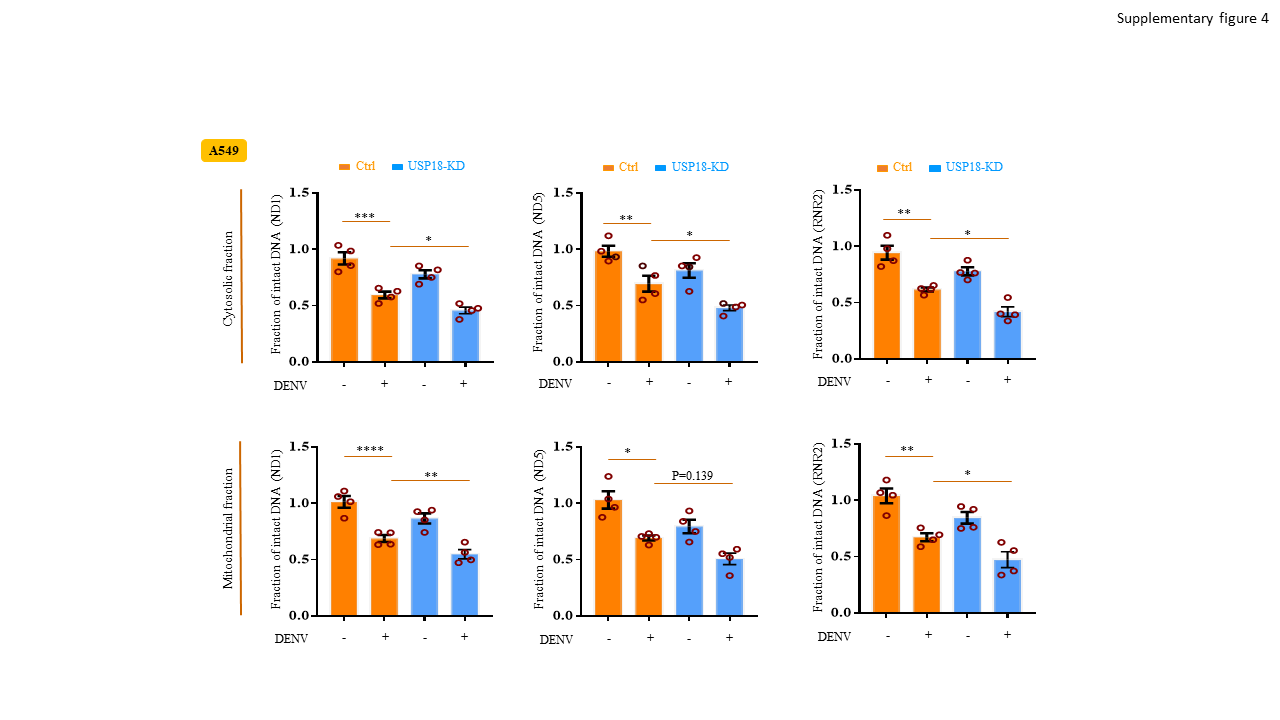


**Supplementary figure 4. USP18 caused oxidative damage of mtDNA**

A549 cells (2 x 10^5^/ml) transfected with siCtl or siUSP18 were infected with mock or DENV (MOI=0.5) for 24 h were collected. The measurement of oxidative damage of mtDNA with formamidopyrimidine DNA glycosylase (Fpg)-sensitive qPCR analysis was performed according to the Materials and Methods. Data were calculated by qPCR as the quotient of signal intensities in Fpg-treated DNA relative to Fpg-untreated DNA and reflect the fraction of intact DNA. More than 3 independent experiments were carried out and analyzed. Statistical analysis was done using two-way ANOVA with Holm-Sidak multiple comparisons to compare differences among different treatments. *, P < 0.05; **, P < 0.01; ***, P < 0.001 and ****, P < 0.0001.


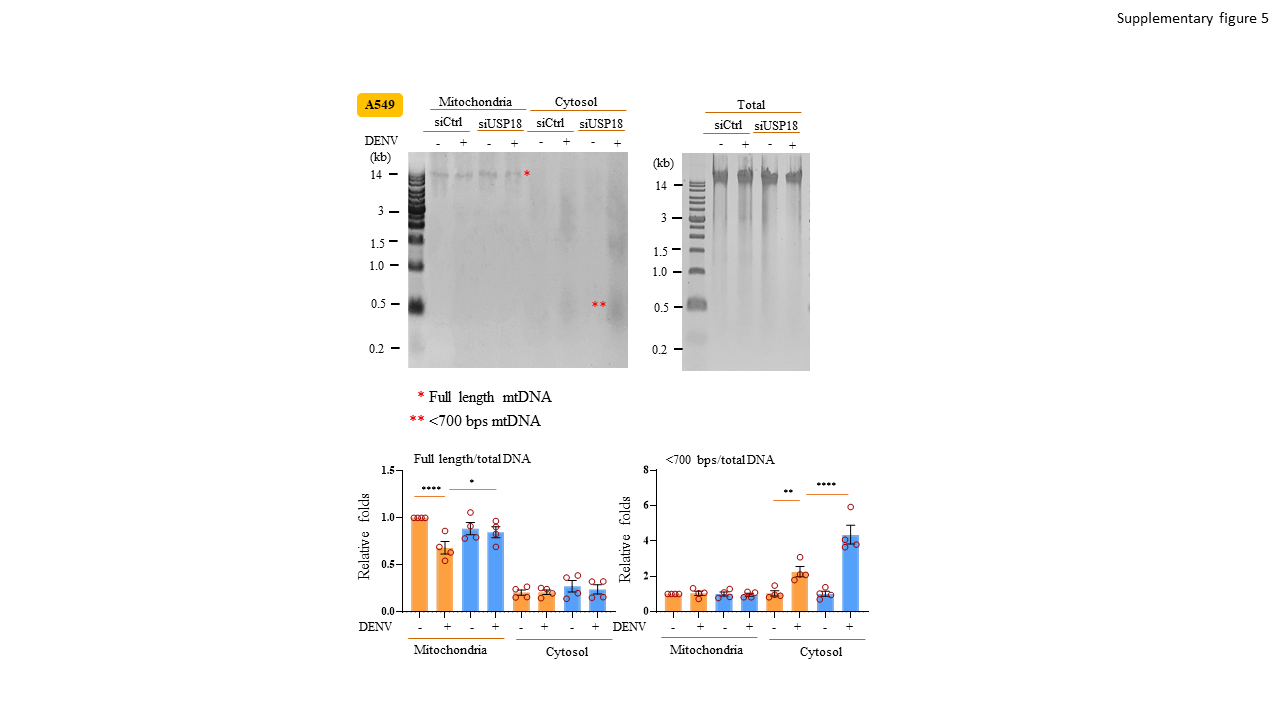


**Supplementary figure 5. USP18 regulated mtDNA fragmentation**

A549 cells (2 x 10^5^/ml) treated with siCtrl or siUSP18 via lipofectamine 3000 were infected with mock or DENV (MOI=0.5) for 24 h. Then DNA was prepared from different fractions of treated cells as described in the Materials and Methods and run in agarose gels and analyzed by staining with Midori Green Advance Safe DNA/RNA staining kit. Quantification for total mtDNA or <700 bp fragments was relative to total DNA intensity. Statistical analysis was done using two-way ANOVA with Holm-Sidak’s multiple comparisons to compare differences among different treatments. *, P < 0.05; **, P < 0.01; ***, P < 0.001 and ****, P < 0.0001. The original gels were presented in Supplementary figure 15.


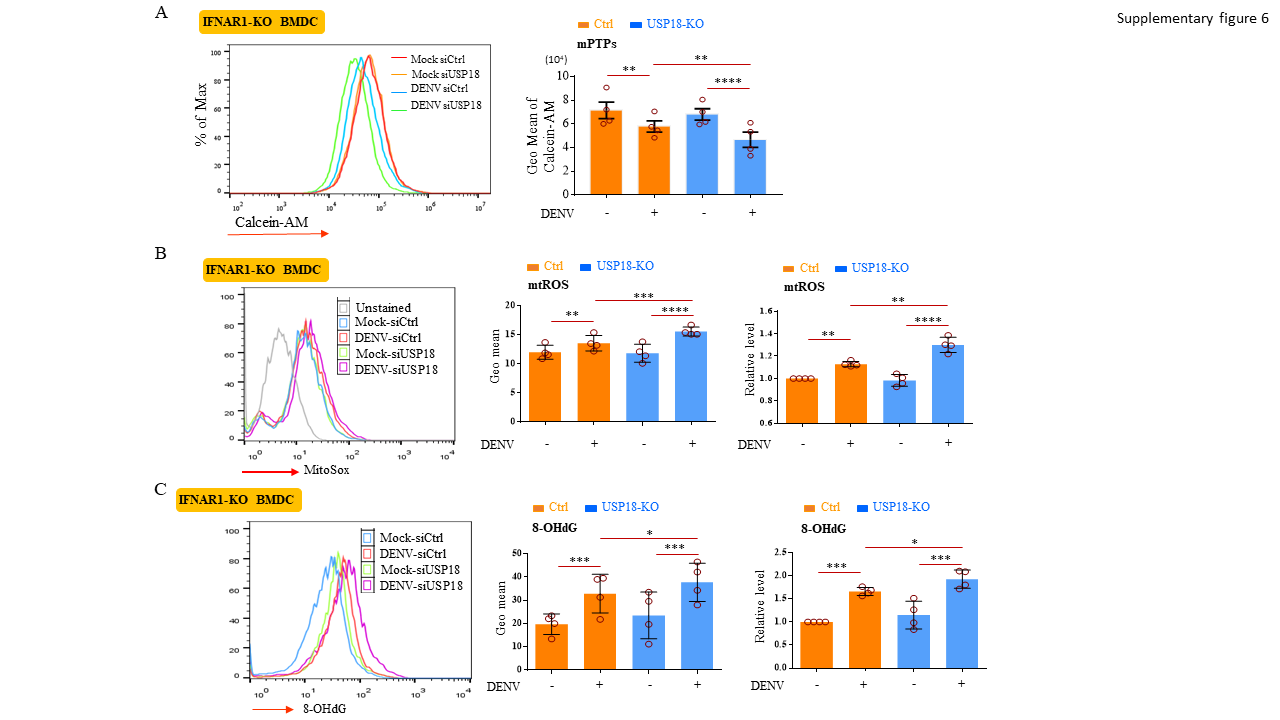


**Supplementary figure 6. USP18 regulated DENV-induced activation of mPTPs opening, mtROS and mtDNA oxidation in IFNAR1-KO BMDC**

BMDCs (1 x 10^6^/ml) transfected with siCtl or siUSP18 (300 nM) were infected with mock or DENV (MOI=1) for 24 h. In calcein-quenching assay, the treated cells were mixed with 1 μM calcein-AM and 1 mM CoCl_2_ and the fluorescent intensity was determined by flow cytometry (A). The levels of mtROS were determined by staining with MitoSox (5 μM) and analyzed with flow cytometry (B). The intracellular immunostaining was done with anti-8OHdG Ab (1:500) and analysis by flow cytometry (C). More than 3 independent experiments were carried out and analyzed. Statistical analysis was done using two-way ANOVA with Holm-Sidak’s multiple comparisons (A-C) to compare differences among different treatments. *, P < 0.05; **, P < 0.01; ***, P < 0.001 and ****, P < 0.0001.


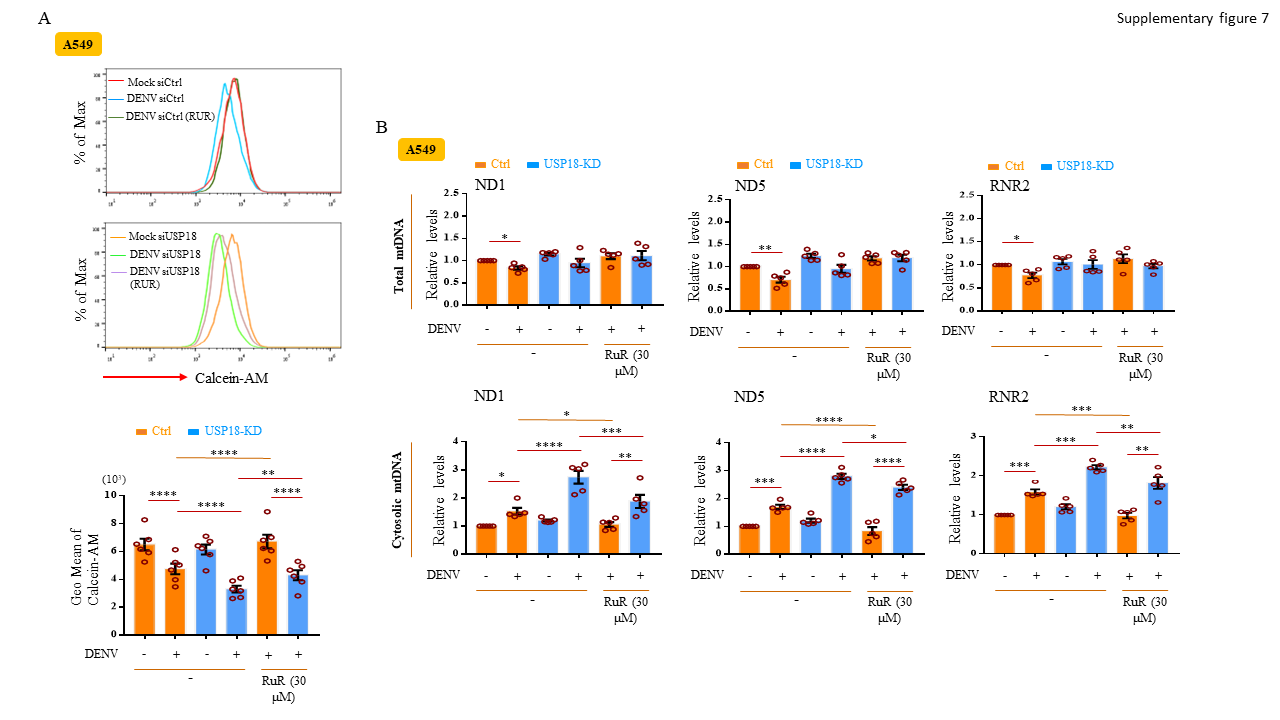


**Supplementary figure 7. USP18 regulated mtDNA release**

A549 cells (2 x 10^5^/ml) transfected with siCtl or siUSP18 were pretreated or not with RuR (30 μM) and then infected with mock or DENV (MOI=0.5) for 24 h. In calcein-quenching assay, the cell lysates prepared from treated cells were mixed with 10 nM calcein and 400 μM CoCl_2_ and the fluorescent intensity was determined by flow cytometry (A). Both total DNA and cytosolic DNA were extracted and quantified using qPCR with specific primers to measure the mtDNA levels. The relative abundance of cytosolic mtDNA levels was determined by normalization with the exogenously added 20 ng of a purified plasmid encoding FLAG gene (PCR3.1-flag) as described in experimental procedures. The total mtDNA content was normalized to nuclear DNA (TERT) (B). More than 3 independent experiments were carried out and analyzed. Statistical analysis was done using two-way ANOVA with Holm-Sidak’s multiple comparisons (A and B) to compare differences among different treatments. *, P < 0.05; **, P < 0.01; ***, P < 0.001 and ****, P < 0.0001.


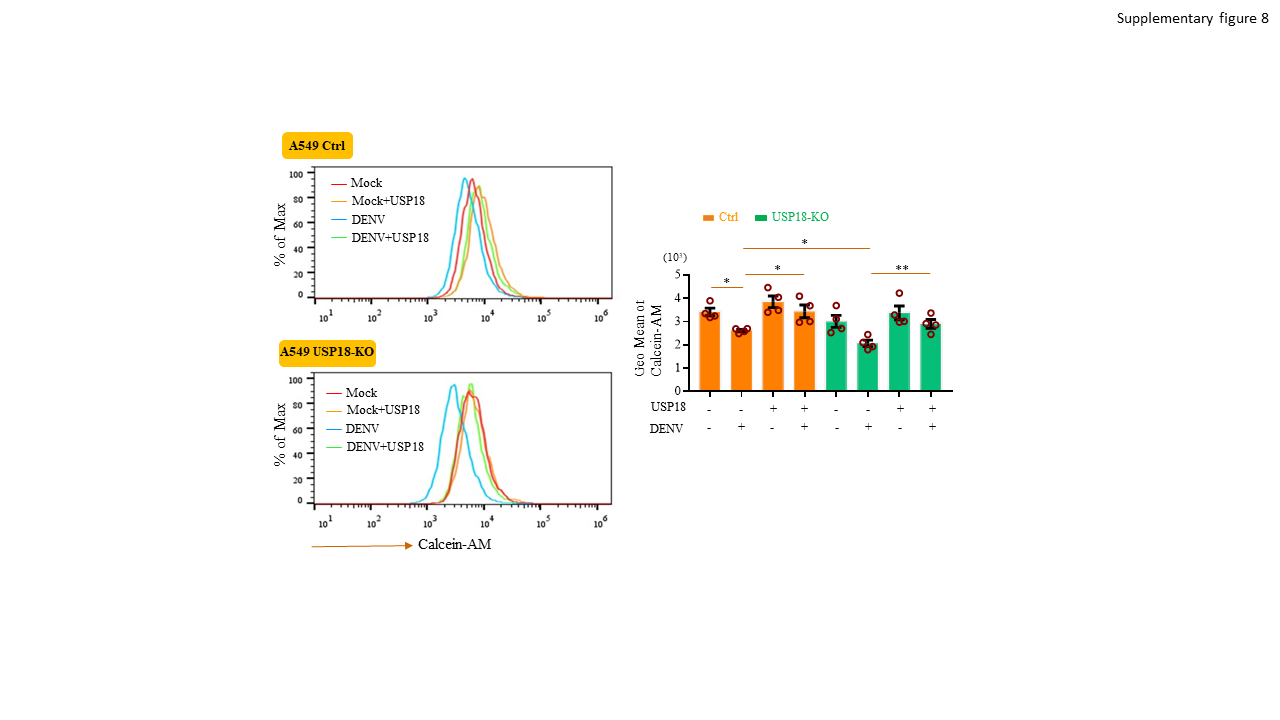


**Supplementary figure 8. Overexpression of USP18 increased DENV replication in USP18-KO cells**

A549 cells (2 x 10^5^/ml) with or without KO of USP18 were transfected to induce overexpression of USP18 and then infected by mock or DENV (MOI=0.5). In calcein-quenching assay, the treated cells were mixed with 10 nM calcein and 400 μM CoCl_2_ and the fluorescent intensity was determined by flow cytometry. More than 3 independent experiments were carried out and analyzed. Statistical analysis was done using two-way ANOVA with Holm-Sidak’s multiple comparisons to compare differences among different treatments. *, P < 0.05; **, P < 0.01; ***, P < 0.001 and ****, P < 0.0001.


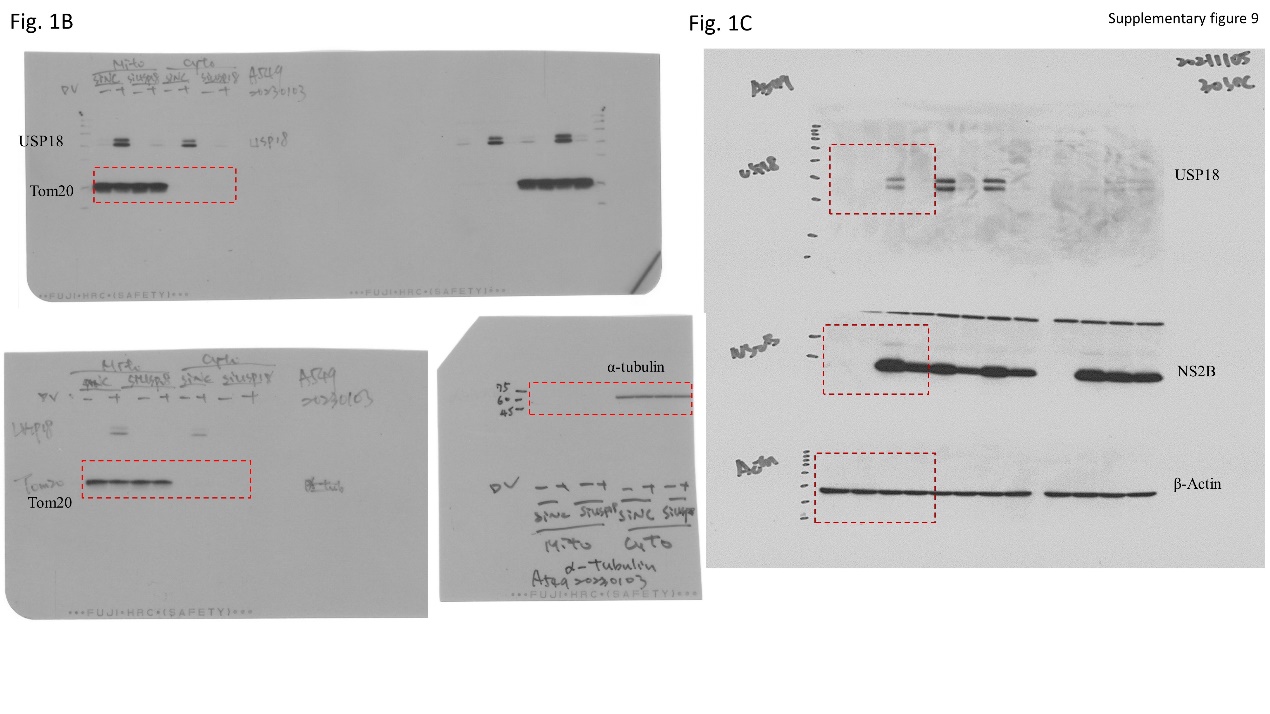


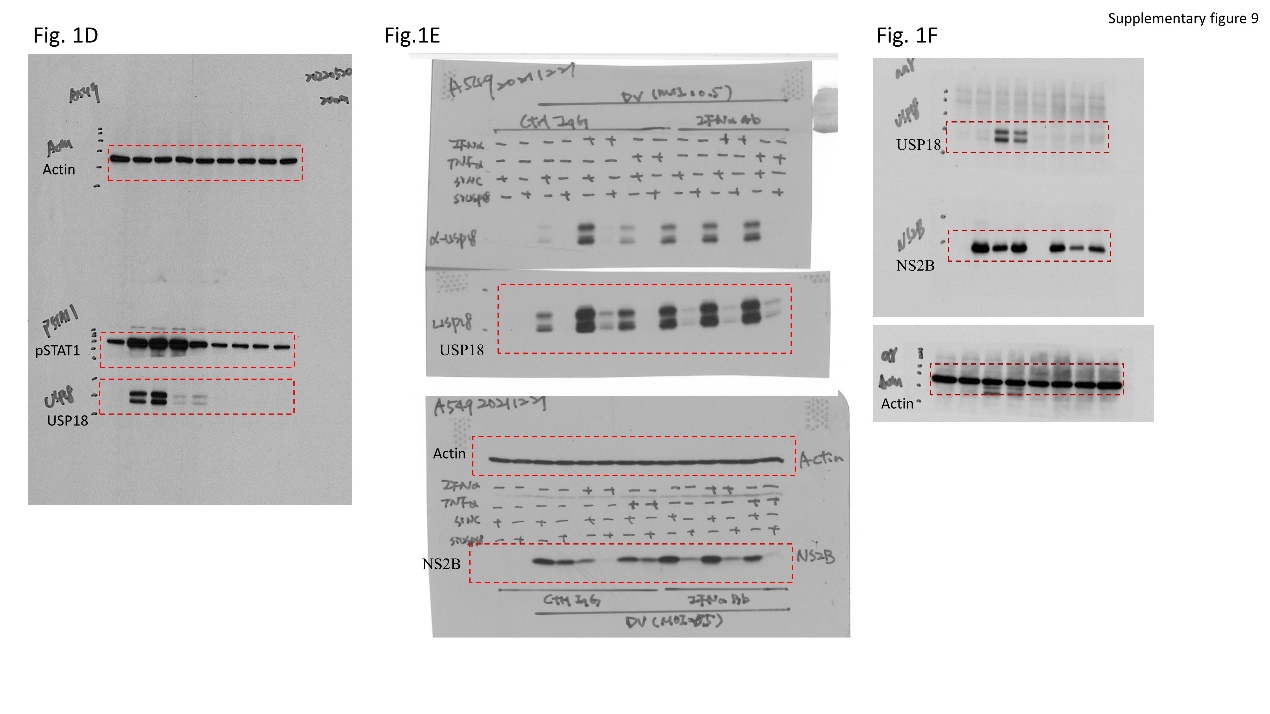


**Supplementary figure 9.** Original gels for Figure 1.


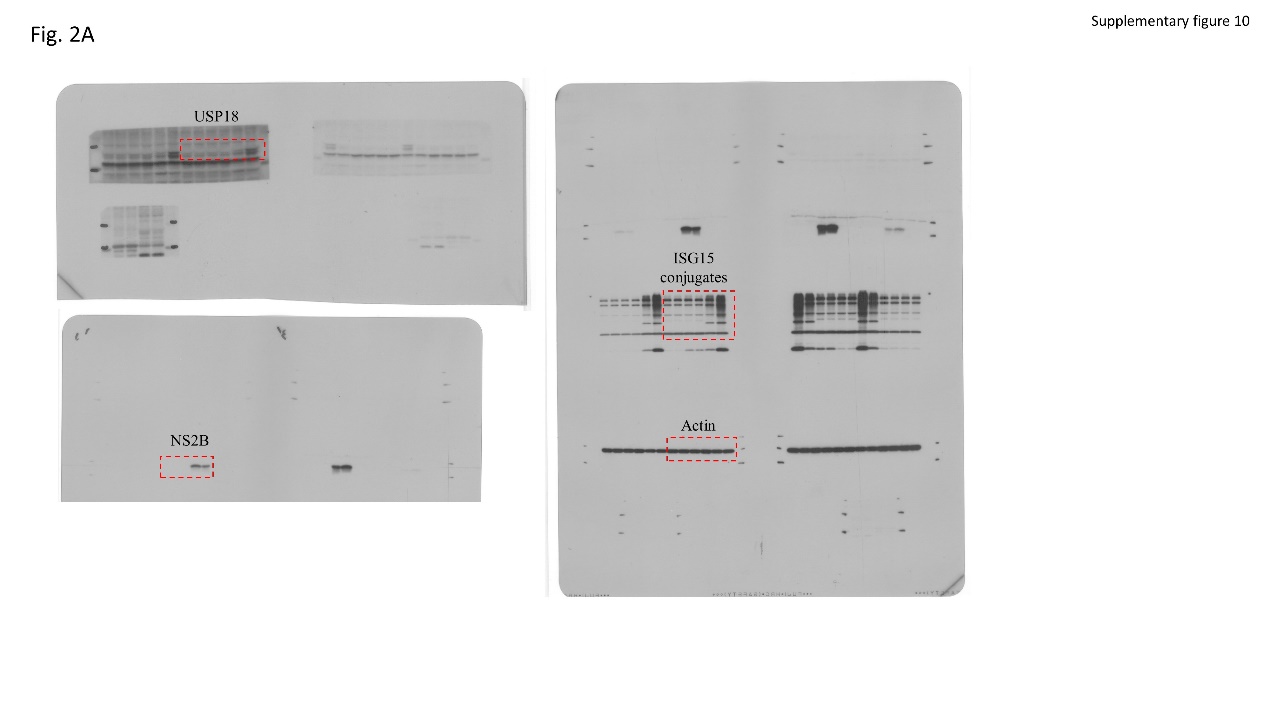


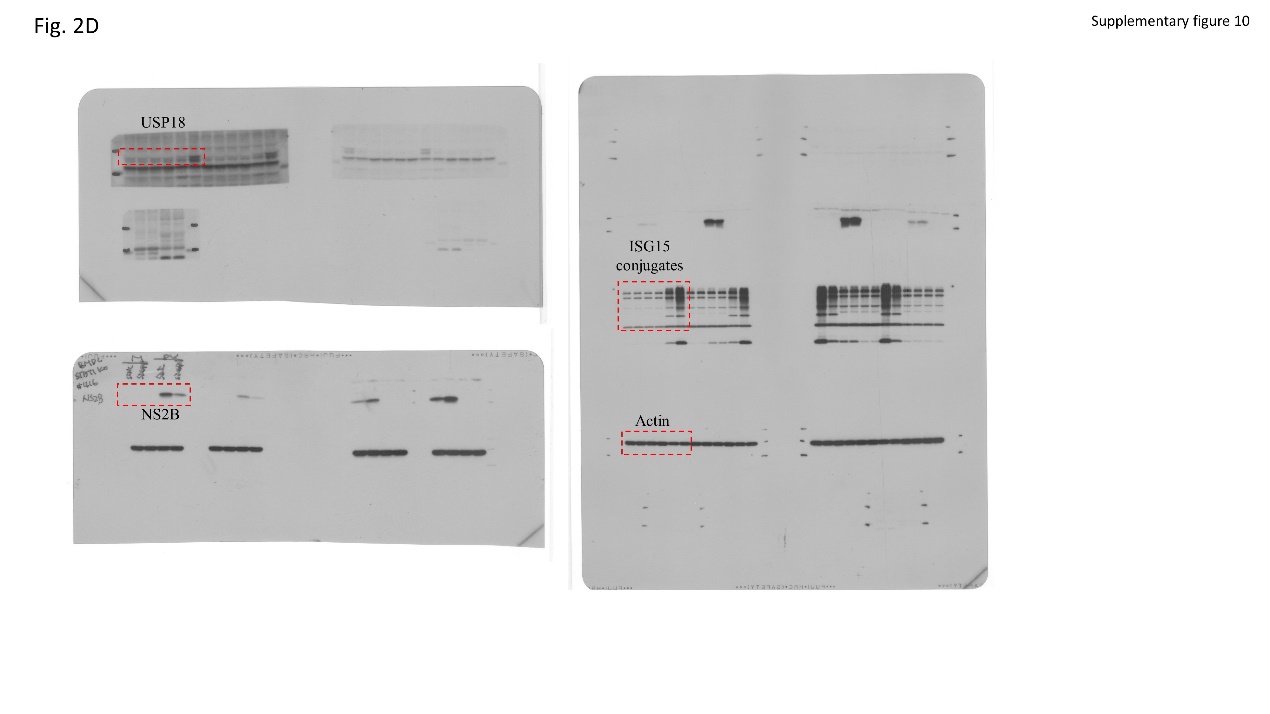


**Supplementary figure 10.** Original gels for Figure 2.


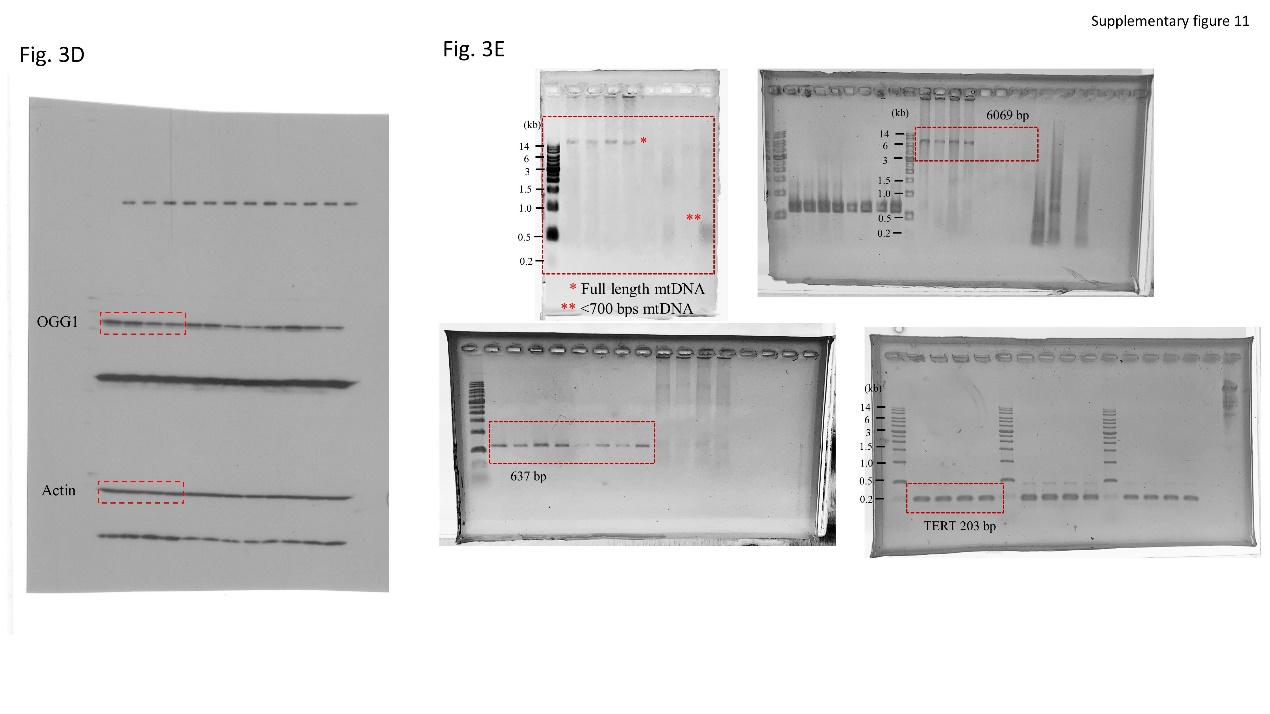


**Supplementary figure 11.** Original gels for Figure 3.


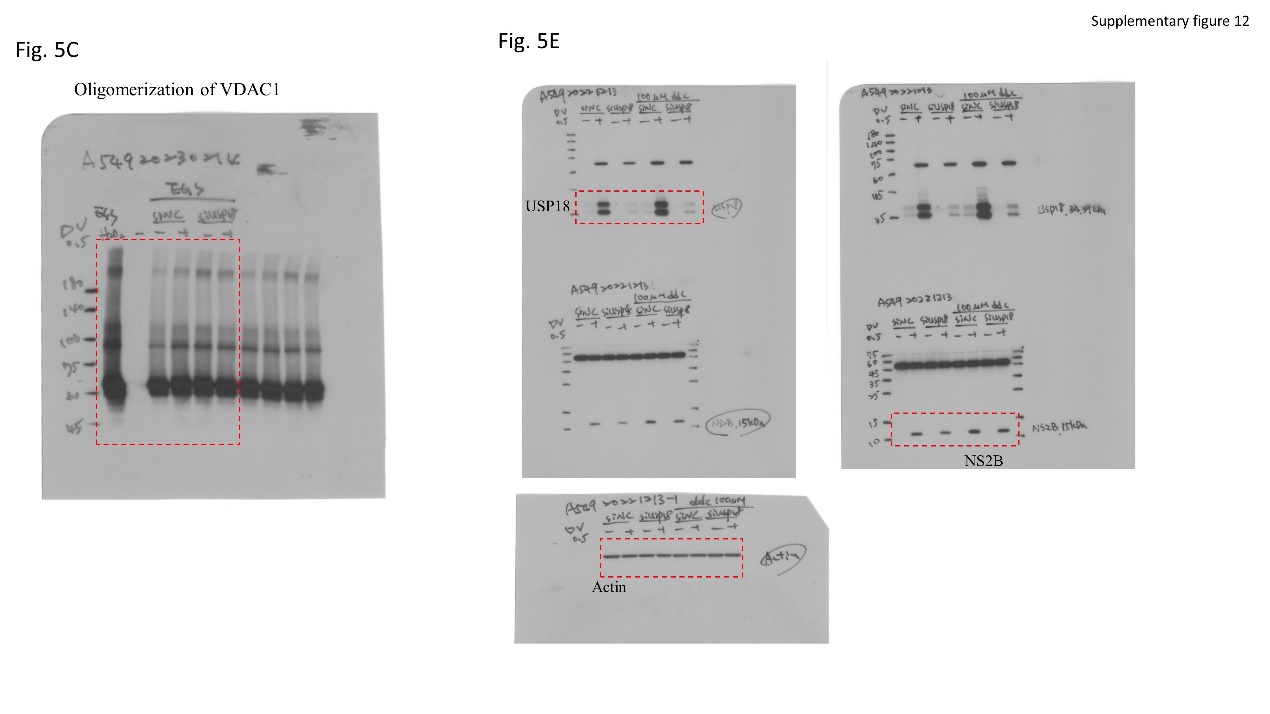


**Supplementary figure 12.** Original gels for Figure 5.


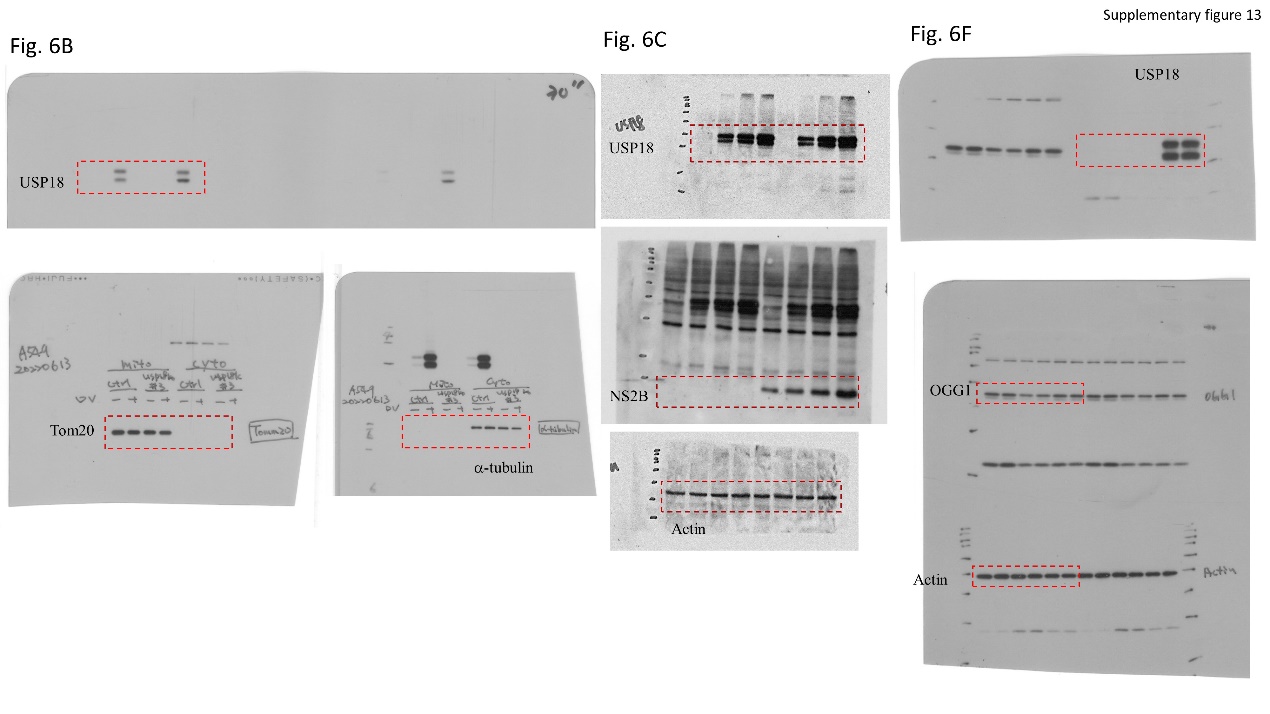


**Supplementary figure 13.** Original gels for Figure 6.


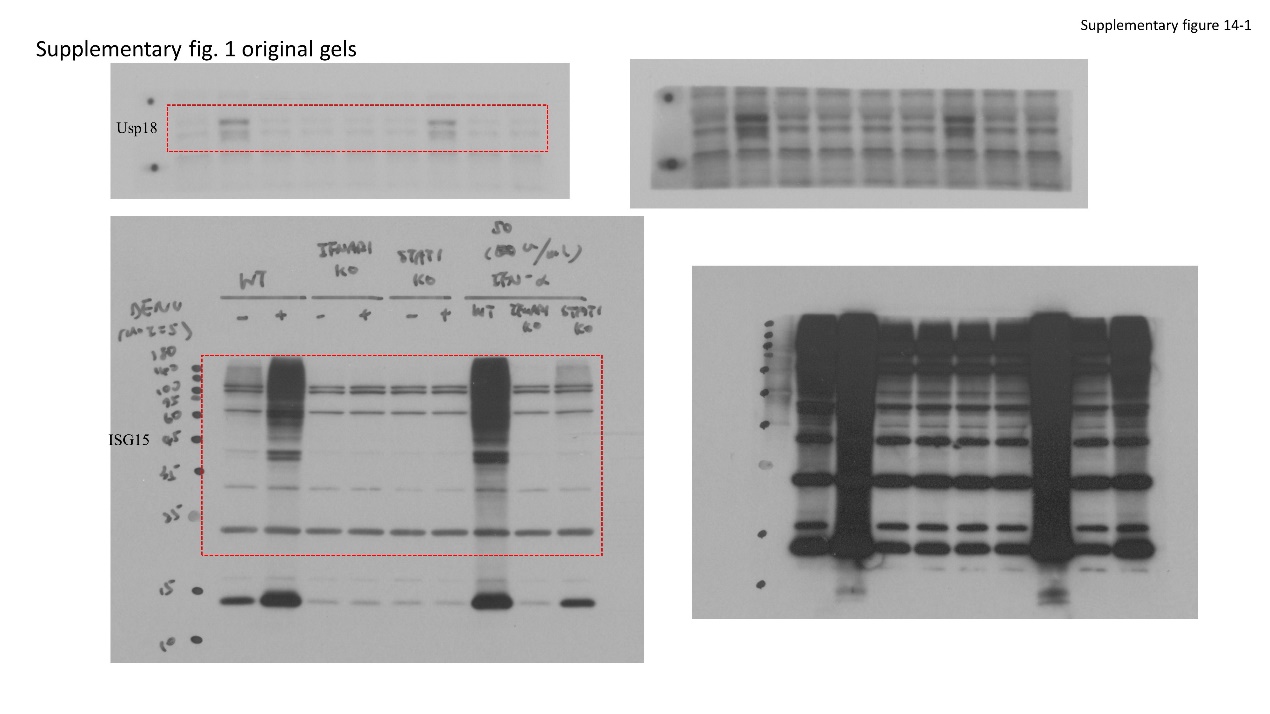


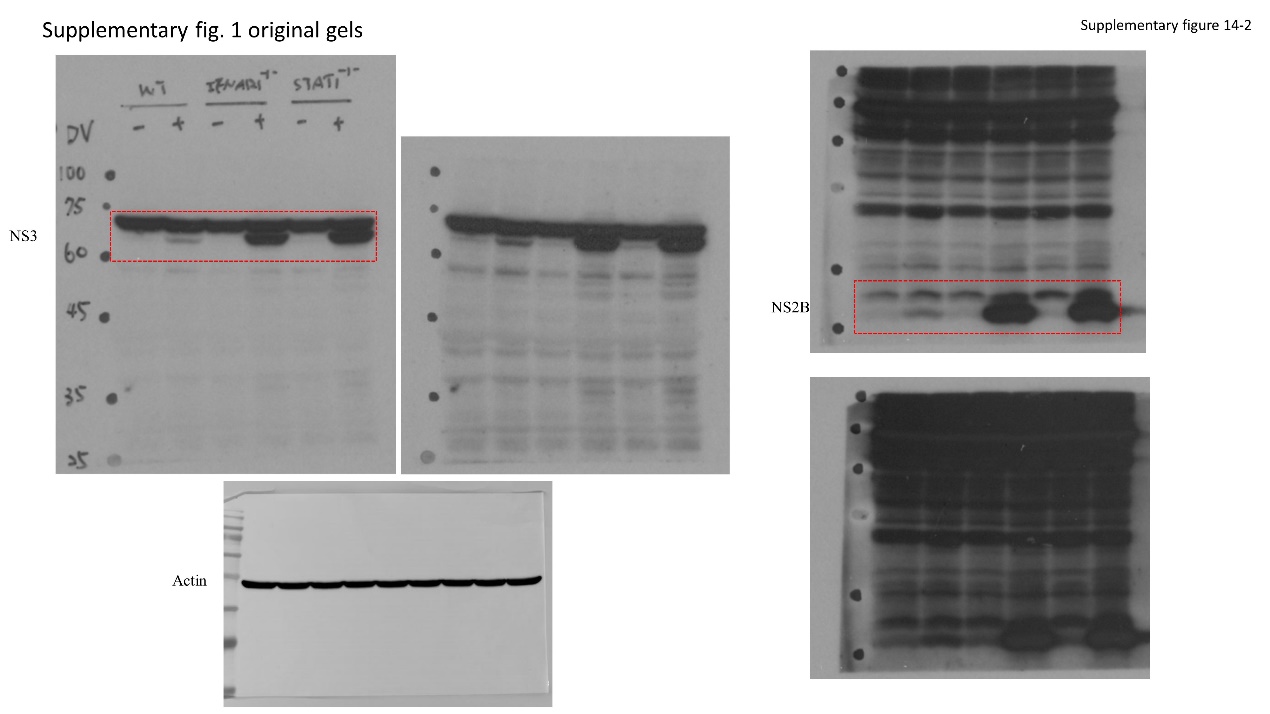


**Supplementary figure 14.** Original gels for Supplementary Figure 1.


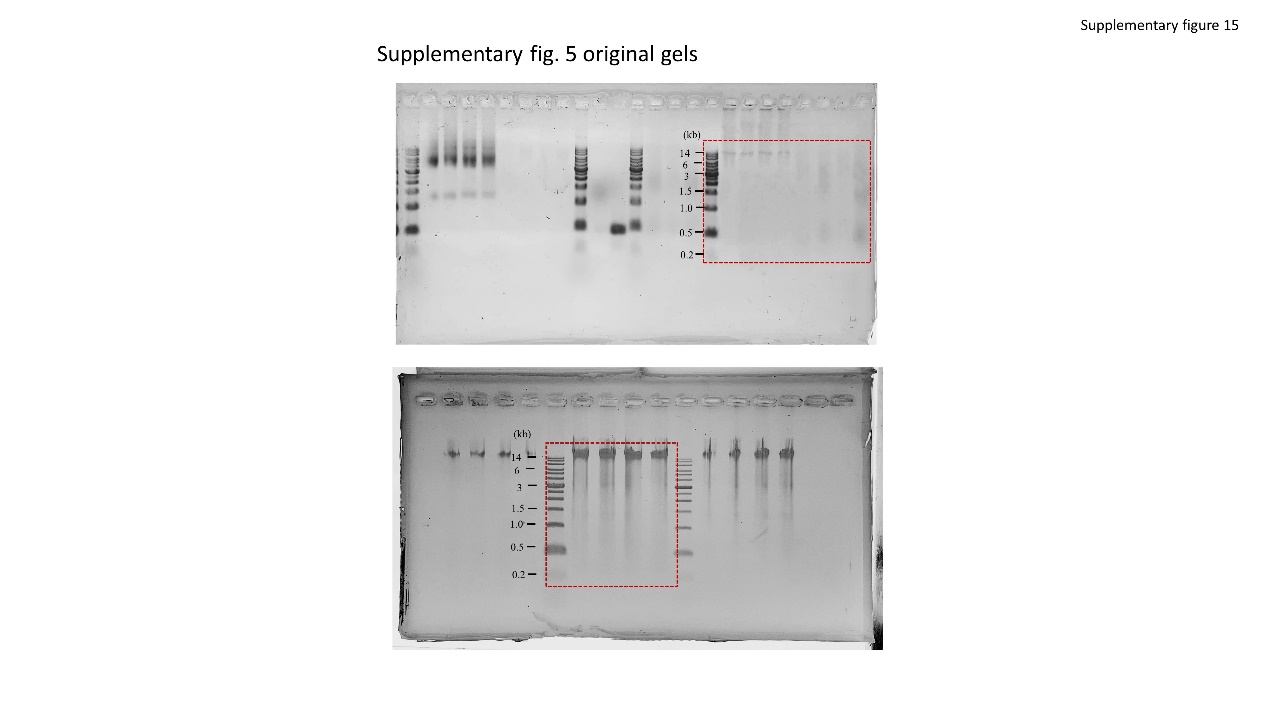


**Supplementary figure 15.** Original gels for Supplementary Figure 5.
